# Supplementary material for: Characterization of an Omnitrap-Orbitrap Platform Equipped with Infrared Multiphoton Dissociation, Ultraviolet Photodissociation, and Electron Capture Dissociation for the Analysis of Peptides and Proteins
Source: Anal Chem. 2023 Aug 3;95(32):12039–46. doi: 10.1021/acs.analchem.3c01899 (PMC10433246; doi:10.1021/acs.analchem.3c01899)
Supplement: Supplementary file 1 — ac3c01899_si_001.pdf [file ac3c01899_si_001.pdf]

Supporting Information

Characterisation of an Omnitrap-Orbitrap platform equipped with IRMPD, UVPD and ExD for the analysis of peptides and proteins

Athanasios Smyrnakis[1‡], Nikita Levin[2,3‡], Mariangela Kosmopoulou[1], Ajay Jha [2,3], Kyle Fort[4], Alexander Makarov[4], Dimitris Papanastasiou[1], Shabaz Mohammed[2,5,6*]

[1] Fasmatech Science & Technology, Lefkippos Tech. Park, NCSR Demokritos, 15341 Agia Paraskevi, Greece
[2] Rosalind Franklin Institute, Harwell Campus, OX11 0QX Didcot, United Kingdom
[3] Department of Pharmacology, University of Oxford, OX1 3QT Oxford, United Kingdom
[4] Thermo Fisher Scientific, 28199 Bremen, Germany
[5] Department of Biochemistry, University of Oxford, OX1 3QU Oxford, United Kingdom
[6] Department of Chemistry, University of Oxford, OX1 3TA Oxford, United Kingdom
[‡] A.S. and N.L. contributed equally

* Email: shabaz.mohammed@chem.ox.ac.uk

Content

		Page No.
Figure S1(A)	Schematic drawing of the Orbitrap-Omnitrap instrument	S3
Figure S1(B)	Schematic drawing of the cross-section of the Omnitrap and IR laser beam propagating along the axis	S4
Figure S2	Schematic drawing of segment Q5 and overlap between an ion cloud and an IR laser beam in Q5	S5
Figure S3	Schematic drawing of segments Q7,Q8, Q9 and overlap between an ion cloud and a UV laser beam in Q8	S6
Figure S4	Pressure profile in the Omnitrap following a pulse of buffer gas	S7
Figure S5	Dependence of IRMPD efficiency on the delay between a gas pulse and IR triggering	S8
Figure S6	Dependence of IRMPD efficiency on the *q* value	S9
Figure S7	Dependence of IRMPD efficiency on the length of IR pulse	S10
Figure S8	IRMPD spectra of [Glu-fibrinopeptide B]$^{2+}$ acquired in segments Q2, Q5 and Q8	S11
Figure S9(A,B,C)	IRMPD spectra of ubiquitin^{8+}, myoglobin^{10+} and [carbonic anhydrase]$^{20+}$ acquired in segment Q5 of the Omnitrap and corresponding fragment mapping	S12
Figure S9(D,E)	Effect of IR laser power on the abundance of internal fragments in IRMPD	S13
Figure S10	Normalised intensities of few selected fragments in the UVPD of [bradykinin]$^{2+}$ for different number of pulses of the UV laser	S14
Figure S11	Normalised TIC of all main-series fragments and normalised intensity of unfragmented precursor in the UVPD of ubiquitin^{8+} for different number of pulses of the UV laser	S15
Figure S12	UVPD spectra of ubiquitin^{8+} and [carbonic anhydrase]$^{20+}$	S16
Figure S13	Number of fragments of different types identified in the UVPD experiments of ubiquitin^{8+}	S17
Figure S14	Fragment maps for UVPD experiments on ubiquitin^{8+}, myoglobin^{10+}, and [carbonic anhydrase]$^{20+}$	S18
Figure S15	UVPD and IR-activated-UVPD spectra of myoglobin^{10+}	S19
Figure S16	Ion currents of fragments of different types and sequence coverages identified in the IR-activated-UVPD experiments of myoglobin^{10+}	S20
Figure S17	UVPD and IR-activated-UVPD spectra of [carbonic anhydrase]$^{20+}$	S21
Figure S18	Ion currents of fragments of different types and sequence coverages identified in the IR-activated-UVPD experiments of [carbonic anhydrase]$^{20+}$	S22
Figure S19	ECD spectra of ubiquitin^{8+}, myoglobin^{20+}, and [carbonic anhydrase]$^{20+}$	S23
Figure S20	Fragment maps of ECD of ubiquitin^{8+}, myoglobin^{20+}, and[carbonic anhydrase]$^{20+}$	S24
Figure S21	Fragment maps of ECD and IR-activated-ECD of myoglobin^{10+}	S25
Figure S22	Sequence coverages by fragments of different types identified in the ECD and IR-activated-ECD experiments of myoglobin^{10+}	S26
Figure S23	EID and IR-activated-EID spectra of myoglobin^{10+}	S27
Figure S24	Fragment maps of EID and IR-activated-EID of myoglobin^{10+}	S28
Figure S25	Sequence coverages by fragments of different types identified in the EID and IR-activated-EID experiments of myoglobin^{10+}	S29
Figure S26	ECD and IR-activated-ECD spectra of [Glu-fibrinopeptide B]$^{2+}$	S30
Figure S27	Distributions of normalised intensities of all *c* and *z* fragments identified in IR-activated-ECD of [Glu-fibrinopeptide B]$^{2+}$ for different power outputs of the IR laser	S31
Figure S28	Summed ion currents of *a*,*b*,*c*,*y*,*z* fragments identified in IR-activated-ECD of triply charged chain B of insulin for different power outputs of the IR laser	S32
Table S1	Details about analytes used in the experiments	S33
Table S2	Types of fragments searched in the spectra using the in-house software and MS-TAFI	S34

A)

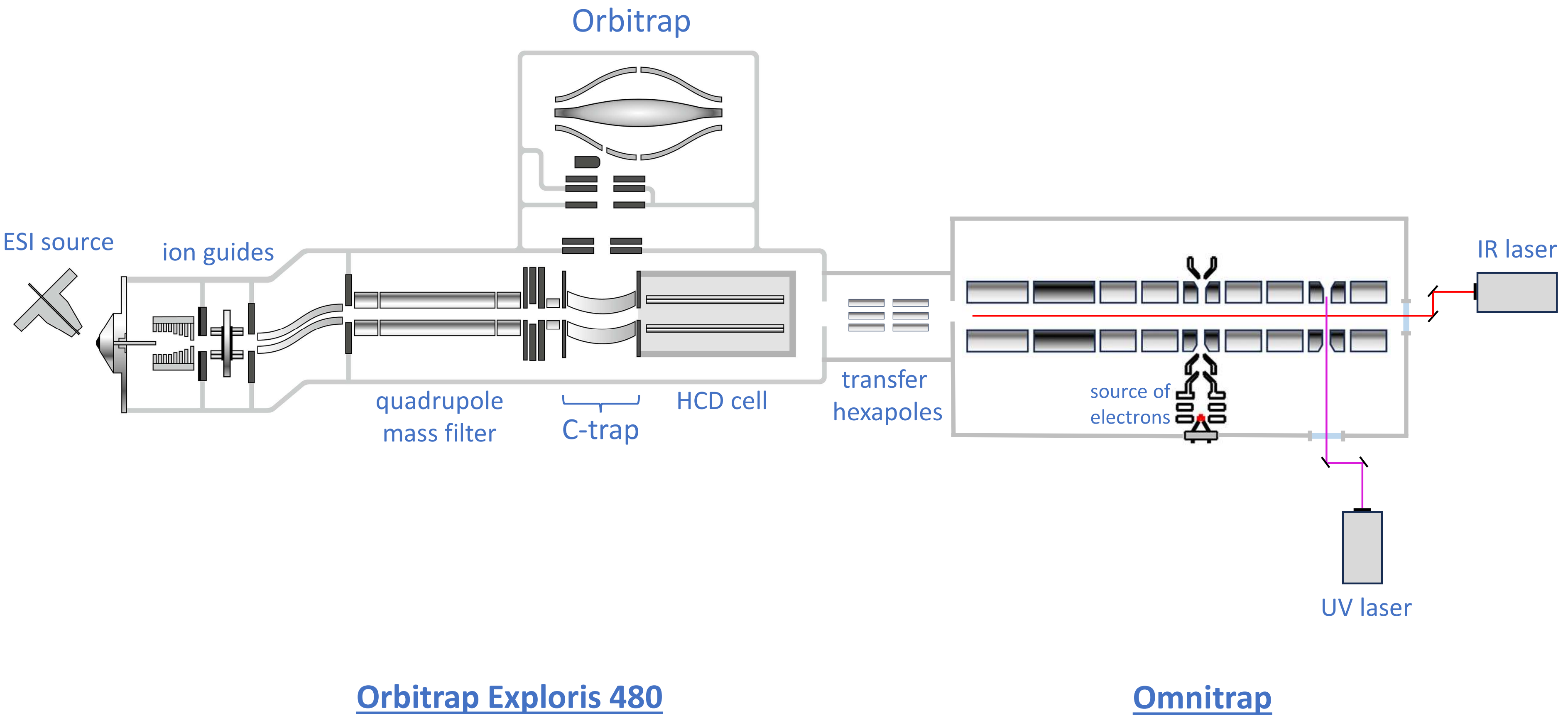

Figure S1. A) Schematic drawing of the Orbitrap-Omnitrap instrument. B) Schematic drawing of the cross-section of the Omnitrap in the plane parallel to its axis and the overlap between an ion cloud (500000 charges) trapped at q=0.15 in Q2/Q5/Q8 and an IR laser beam. The radial density of the ion cloud is represented by a normal distribution with a variance (σ) of ~0.4 mm. The variation corresponding to 4σ covers approximately 99% of the population of charges. The ideal case of a laser beam perfectly aligned along the axis is considered.

B)

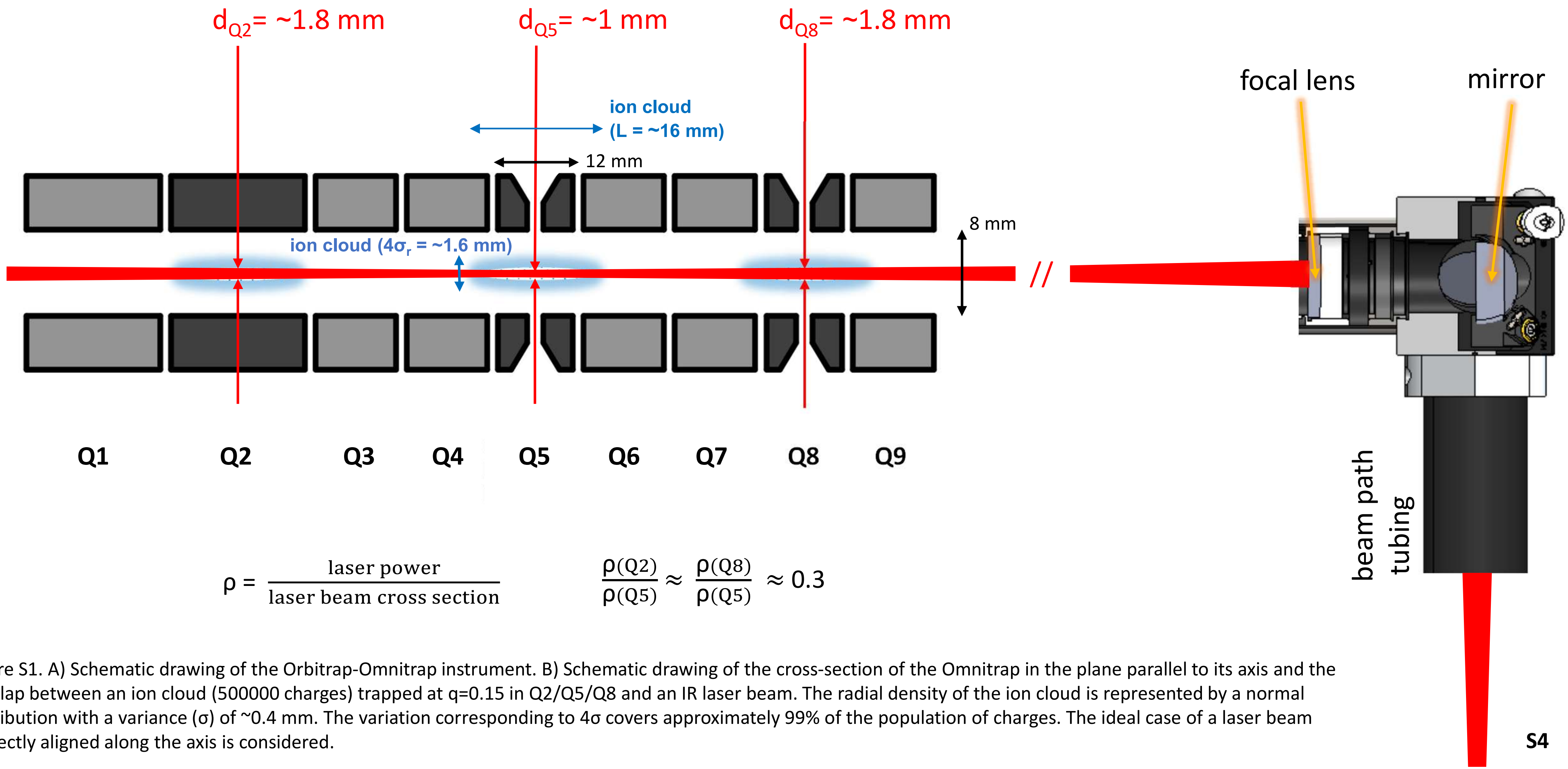

$$\rho = \frac{\text{laser power}}{\text{laser beam cross section}} \qquad \frac{\rho(Q2)}{\rho(Q5)} \approx \frac{\rho(Q8)}{\rho(Q5)} \approx 0.3$$

Figure S1. A) Schematic drawing of the Orbitrap-Omnitrap instrument. B) Schematic drawing of the cross-section of the Omnitrap in the plane parallel to its axis and the overlap between an ion cloud (500000 charges) trapped at q=0.15 in Q2/Q5/Q8 and an IR laser beam. The radial density of the ion cloud is represented by a normal distribution with a variance (σ) of ~0.4 mm. The variation corresponding to 4σ covers approximately 99% of the population of charges. The ideal case of a laser beam perfectly aligned along the axis is considered.

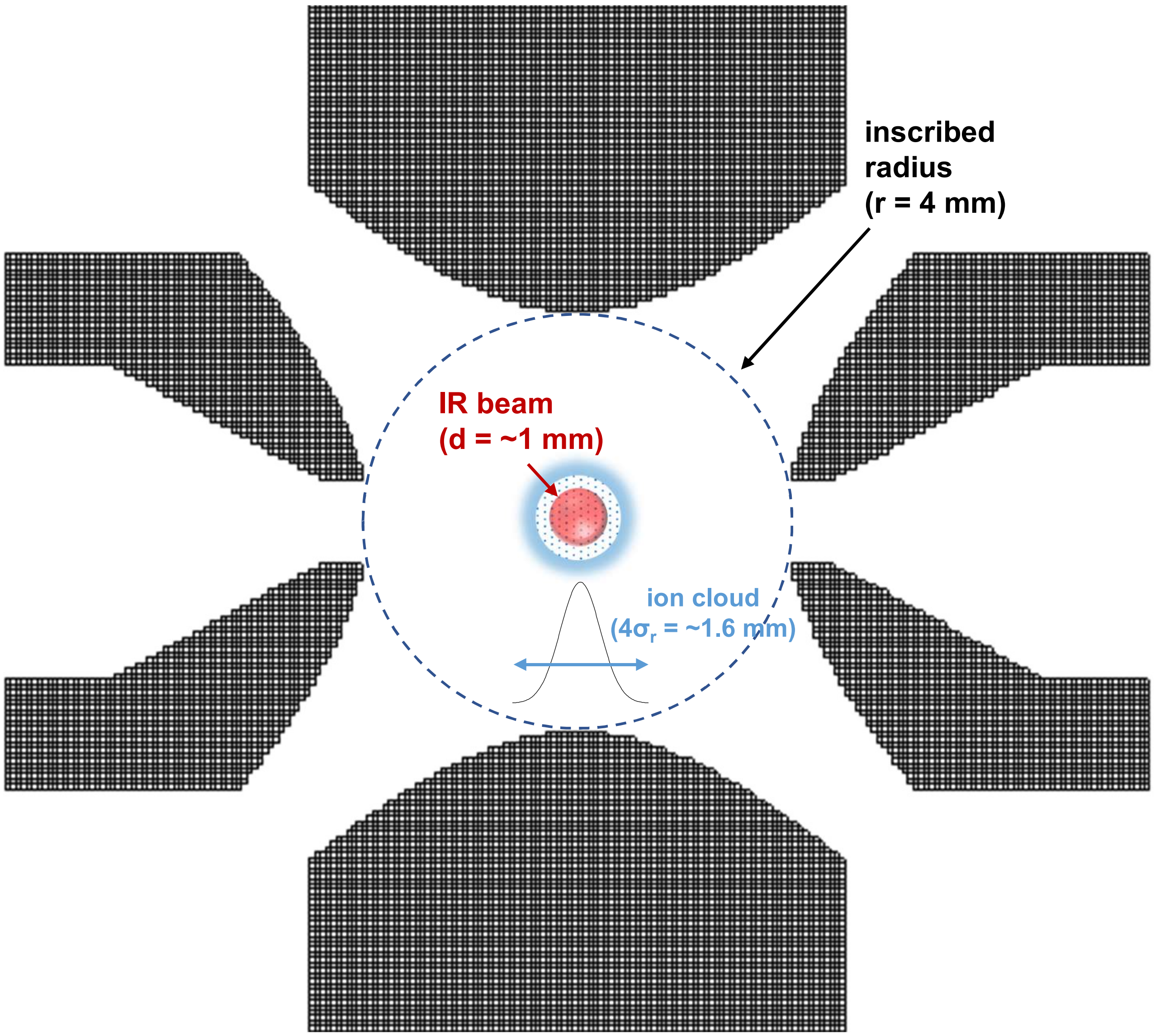

Figure S2. Schematic drawing of the cross-section of the segment Q5 in the plane orthogonal to the axis of the trap and the overlap between an ion cloud (500000 charges) trapped at q=0.15 and an IR laser beam. The radial density of the ion cloud is represented by a normal distribution with a variance (σ) of ~0.4 mm. The variation corresponding to 4σ covers approximately 99% of the population of charges.

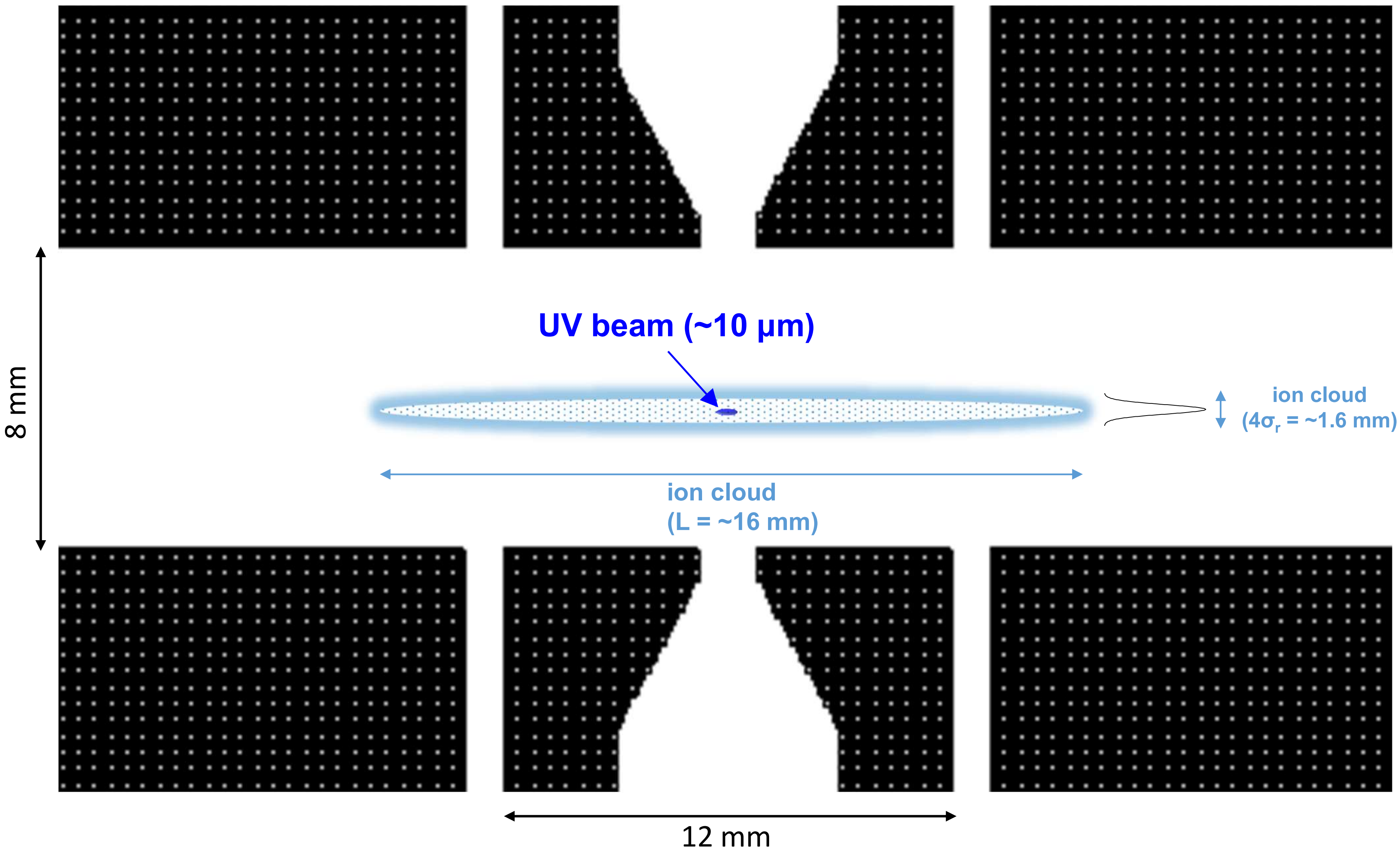

Figure S3. Schematic drawing of the cross-section of the segments Q7, Q8, Q9 in the plane parallel to the axis of the trap and the overlap between an ion cloud (500000 charges) trapped at q=0.15 and a UV laser beam. The radial density of the ion cloud is represented by a normal distribution with a variance (σ) of ~0.4 mm. The variation corresponding to 4σ covers approximately 99% of the population of charges.

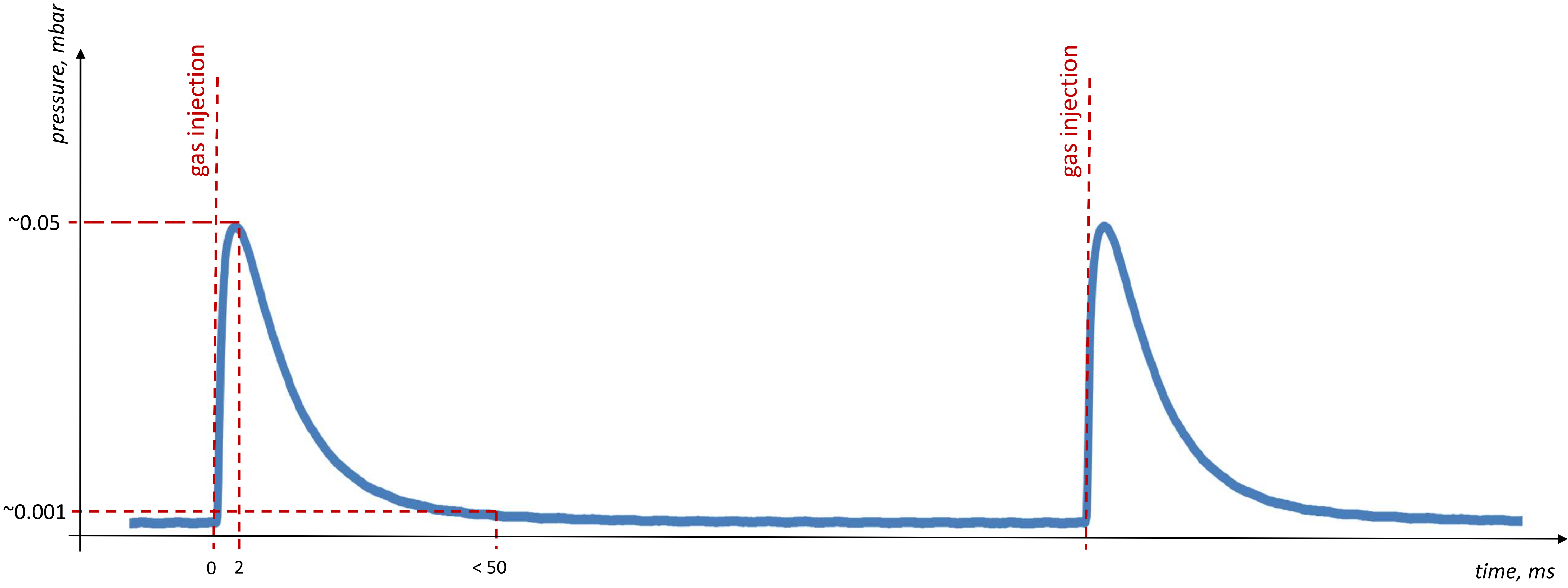

Figure S4. Pressure profile in the Omnitrap following a pulse of buffer gas. The temporal profile of pressure is controlled by adjusting the width of the voltage pulse applied to the solenoid valve. A maximum pressure of 0.05 mbar is reached within 2 ms from releasing nitrogen gas into the ion trapping region, while the period where pressure is sufficiently high to promote collisional cooling (>0.001 mbar) can be extended up to ~50 ms.

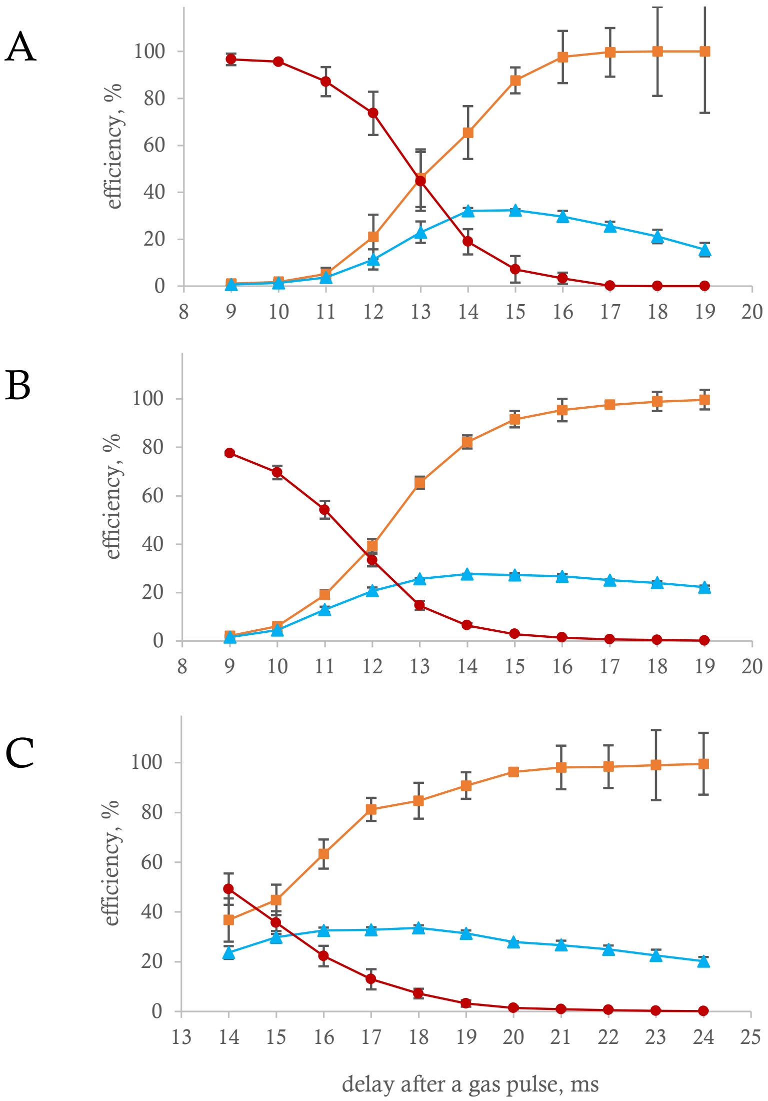

	Q2	Q5	Q8
irrad. time, ms	11	4	12
q value	0.2	0.2	0.2
laser power, % duty cycle	30	30	30

$$\text{photodiss. eff.} = \frac{F}{F + P}$$

$$\text{ion trapping eff.} = \frac{F}{P_0}$$

$$\text{norm. int. of precursor} = \frac{P}{P_0}$$

F – summed intensities of all fragments
P – remaining intensity of precursor after fragmentation
P_0 – intensity of precursor before fragmentation

Figure S5. The dependence of the photodissociation efficiency, trapping efficiency and normalised intensity of the precursor on the delay between a gas pulse and IR triggering in the segments Q2 (A), Q5 (B), and Q8 (C) in the IRMPD of [Glu-fibrinopeptide B]$^{2+}$. The parameters of the experiments are given in the table next to the charts. Each data point represents an average of a triplicate with standard deviations given as error bars.

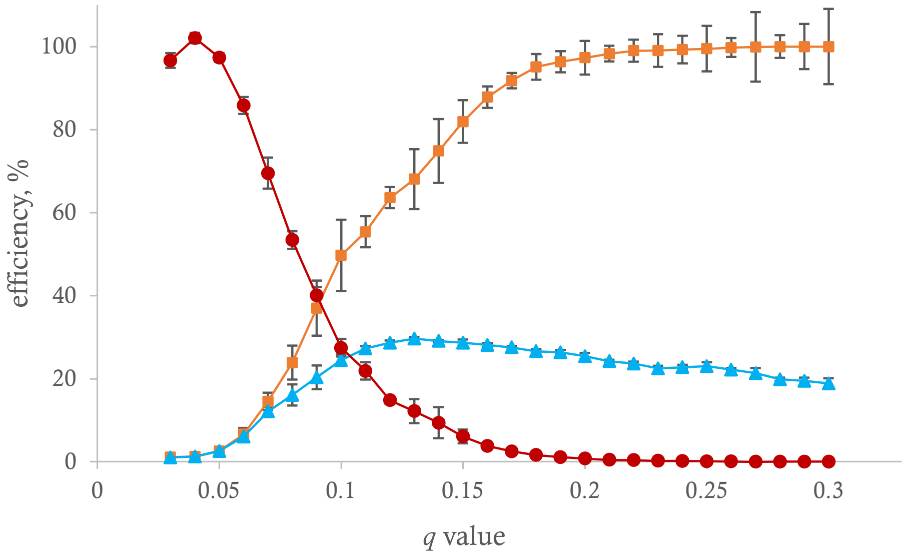

	Q5
irrad. time, ms	5
delay after a gas pulse	13
laser power, % duty cycle	30

$$\text{photodiss. eff.} = \frac{F}{F + P}$$

$$\text{ion trapping eff.} = \frac{F}{P_0}$$

$$\text{norm. int. of precursor} = \frac{P}{P_0}$$

F – summed intensities of all fragments
P – remaining intensity of precursor after fragmentation
P_0 – intensity of precursor before fragmentation

Figure S6. The dependence of the photodissociation efficiency, trapping efficiency and normalised intensity of the precursor on the *q* value of the Omnitrap in the segment Q5 in the IRMPD of [Glu-fibrinopeptide B]$^{2+}$. The parameters of the experiment are given in the table next to the chart. Each data point represents an average of a triplicate with standard deviations given as error bars.

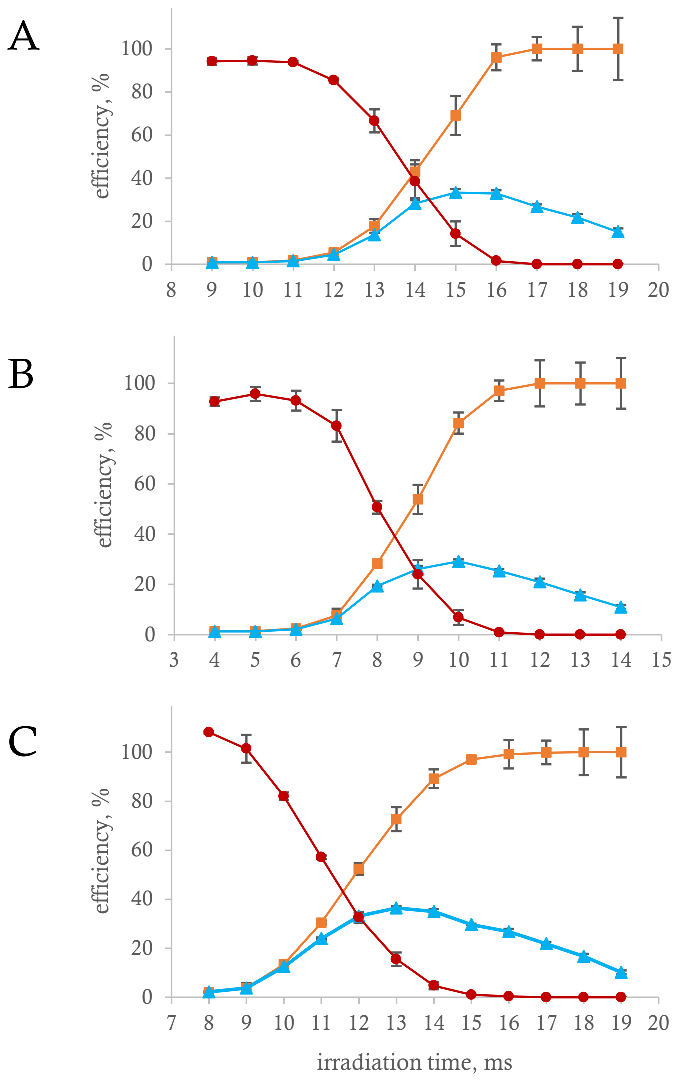

	Q2	Q5	Q8
laser power, %<br>duty cycle	30	20	30
q value	0.2	0.2	0.2
delay after a gas pulse	10	10	15

$$\text{photodiss. eff.} = \frac{F}{F+P}$$

$$\text{ion trapping eff.} = \frac{F}{P_0}$$

$$\text{norm. int. of precursor} = \frac{P}{P_0}$$

F – summed intensities of all fragments
P – remaining intensity of precursor after fragmentation
P_0 – intensity of precursor before fragmentation

Figure S7. The dependence of the photodissociation efficiency, trapping efficiency and normalised intensity of the precursor on the length of IR irradiation in the segments Q2 (A), Q5 (B), and Q8 (C) in the IRMPD of [Glu-fibrinopeptide B]$^{2+}$. The parameters of the experiments are given in the table next to the charts. Each data point represents an average of a triplicate with standard deviations given as error bars.

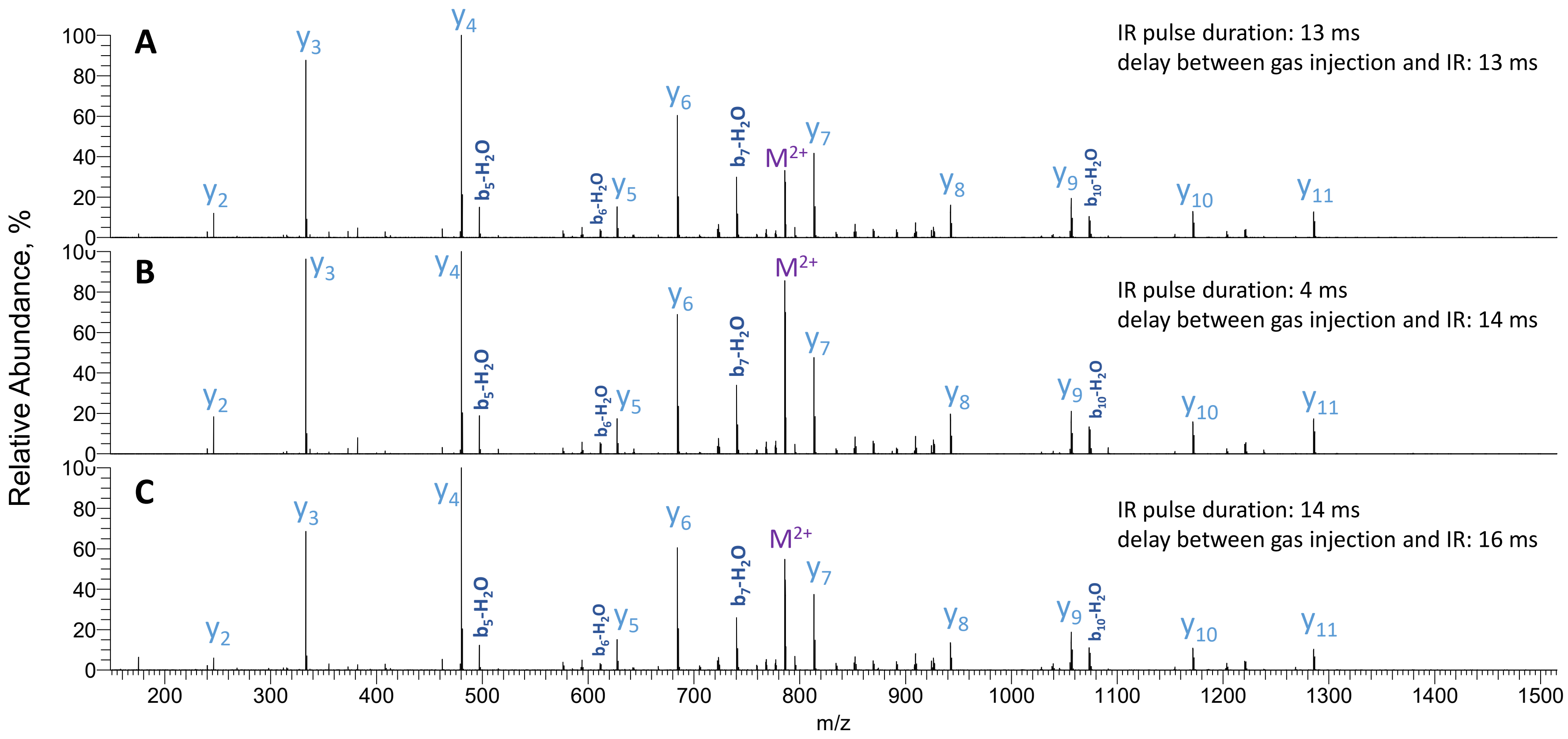

Figure S8. IRMPD spectra of [Glu-fibrinopeptide B]$^{2+}$ acquired in the segments Q2 (A), Q5 (B) and Q8 (C). IRMPD spectra were acquired at q=0.2, 30% laser duty cycle. Laser pulse durations and delays between gas injection and IR pulsing are given in the figure

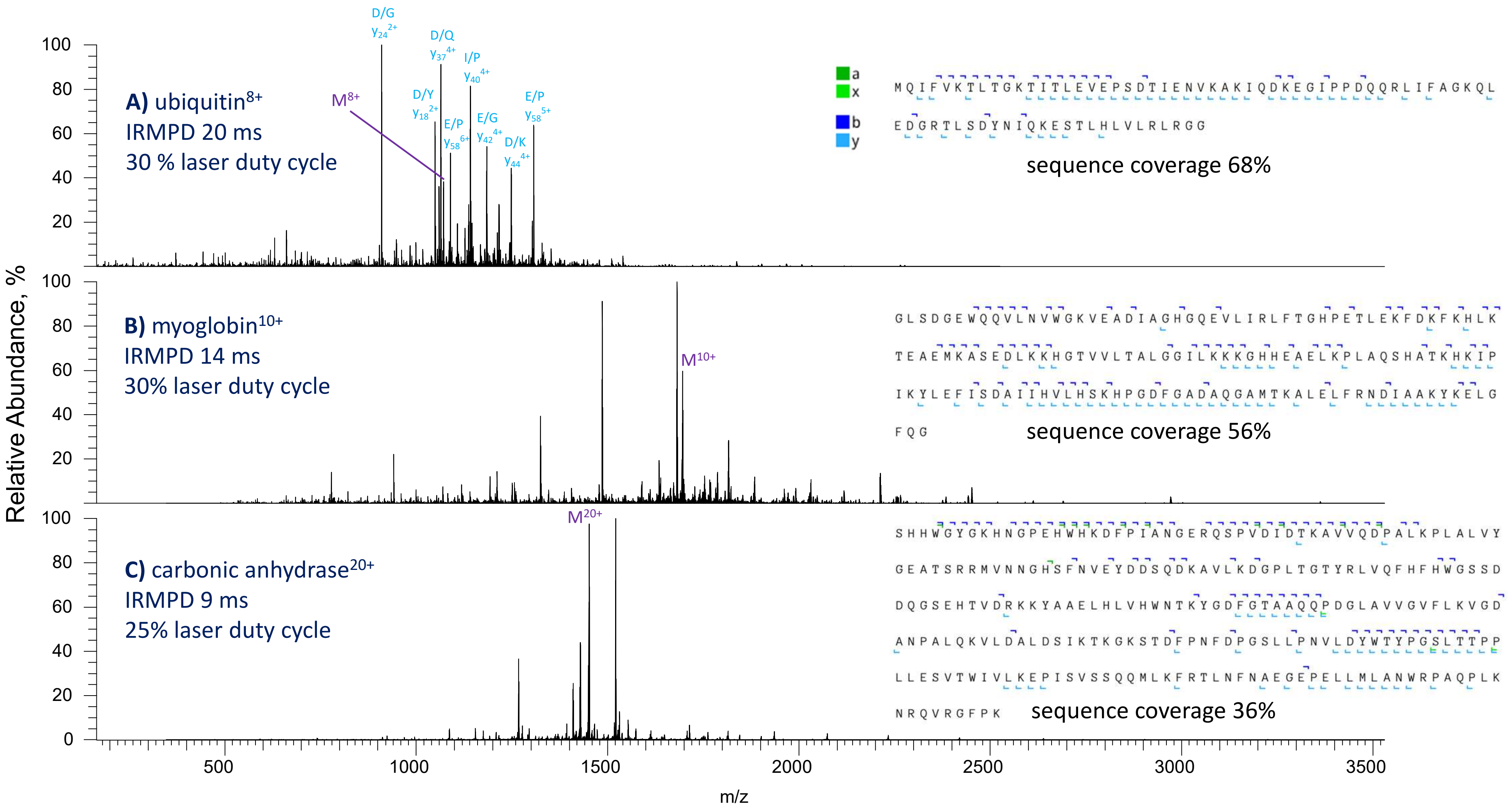

Figure S9. IRMPD spectra of ubiquitin^{8+} (A), myoglobin^{10+} (B) and [carbonic anhydrase]$^{20+}$ (C) acquired in the segment Q5 of the Omnitrap at q=0.2 and corresponding fragment mapping. IRMPD irradiation times and laser powers are given in the figure (panels A, B, C); the IR was triggered 11 ms after the gas pulse. D) IRMPD spectrum of ubiquitin^{8+} acquired in Q5 at q=0.25 and 25% laser duty cycle; E) same experimental conditions as in (D) but 30% laser duty cycle was used. Panels D and E show that increased laser power leads to higher intensities of internal fragments using the example of the singly charged internal fragment (52-57).

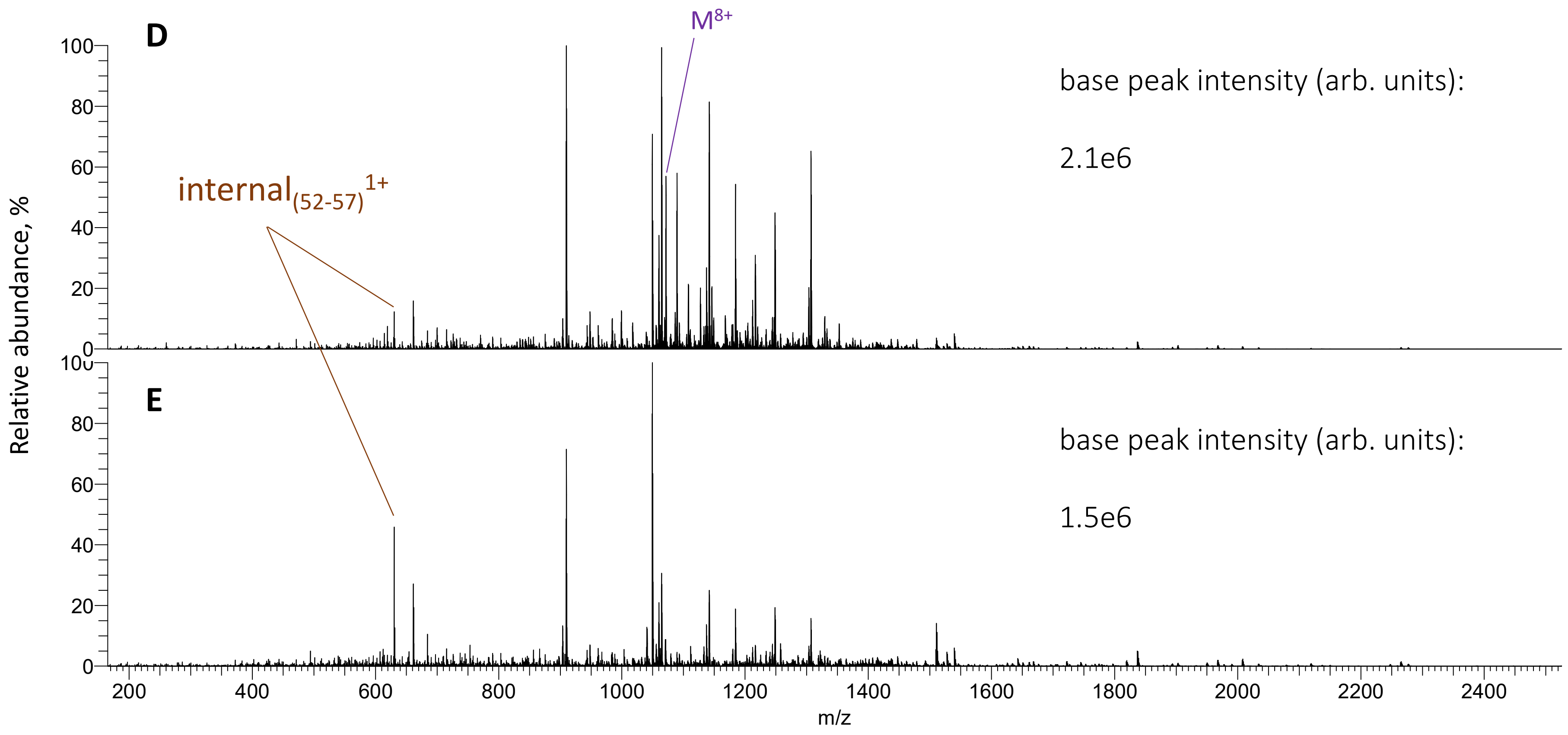

Figure S9. IRMPD spectra of ubiquitin^{8+} (A), myoglobin^{10+} (B) and [carbonic anhydrase]$^{20+}$ (C) acquired in the segment Q5 of the Omnitrap at q=0.2 and corresponding fragment mapping. IRMPD irradiation times and laser powers are given in the figure (panels A, B, C); the IR was triggered 11 ms after the gas pulse. D) IRMPD spectrum of ubiquitin^{8+} acquired in Q5 at q=0.25 and 25% laser duty cycle; E) same experimental conditions as in (D) but 30% laser duty cycle was used. Panels D and E show that increased laser power leads to higher intensities of internal fragments using the example of the singly charged internal fragment (52-57).

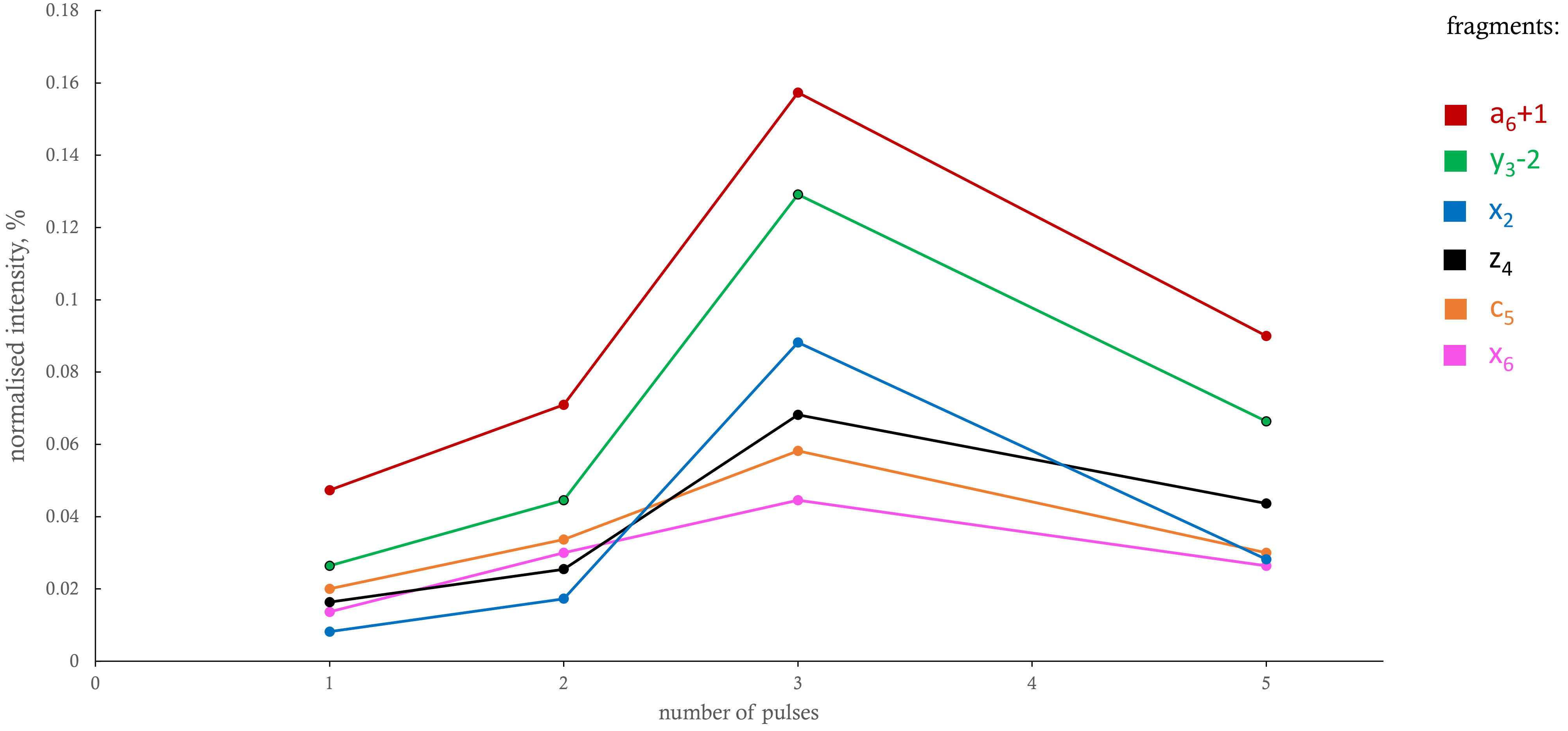

Figure S10. Intensities of few selected fragments normalised to the MS1 intensity of the precursor in the UVPD of [bradykinin]$^{2+}$ for different number of pulses of the UV laser. The energy of each pulse was 5 mJ.

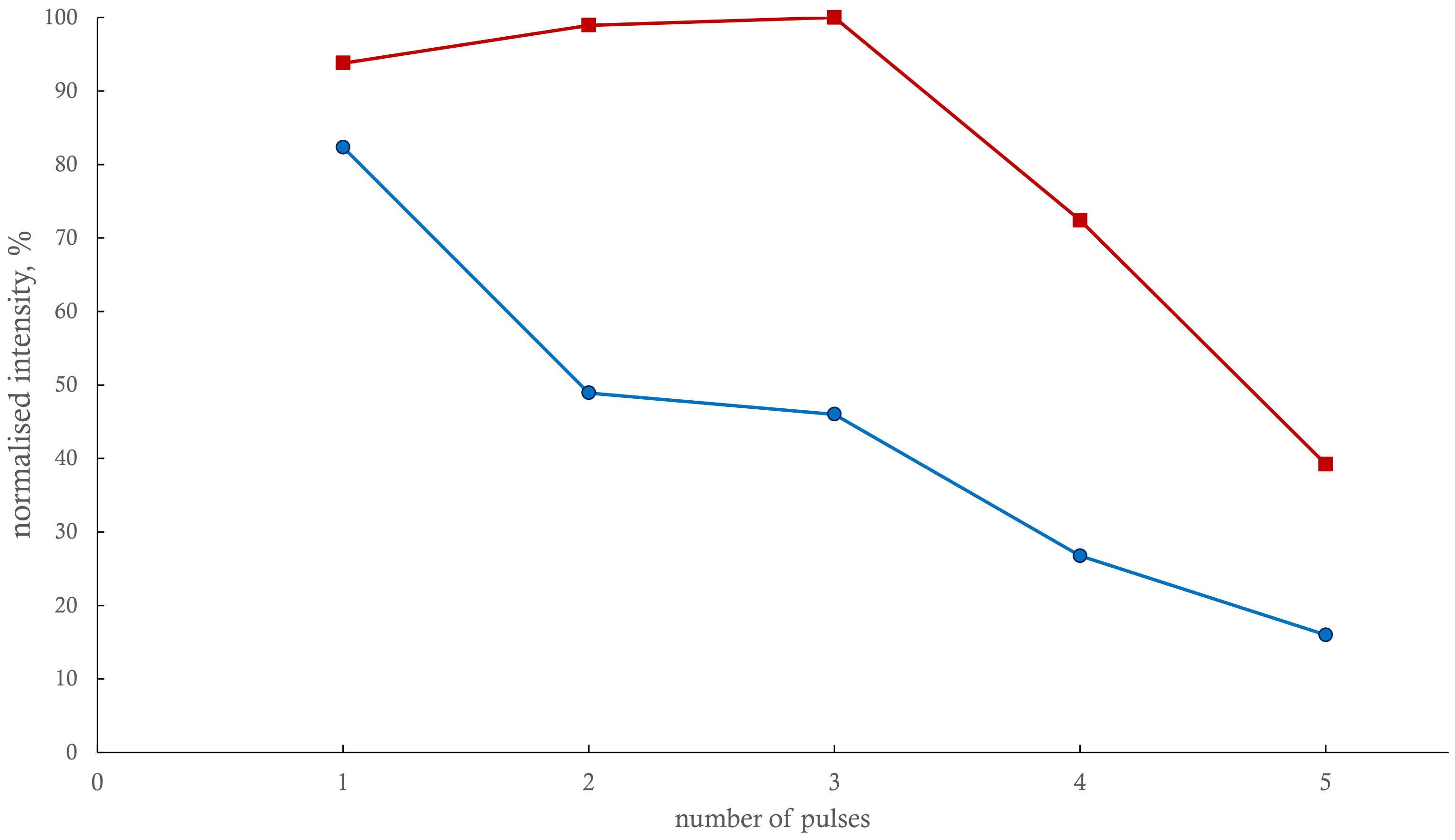

Figure S11. Total ion current of all main-series fragments of ubiquitin^{8+} normalised to the highest value in the series (red) and intensity of unfragmented ubiquitin^{8+} precursor normalised to its intensity in MS1 (blue) in the UVPD experiments for different number of pulses of the UV laser. The energy of each pulse was 5 mJ.

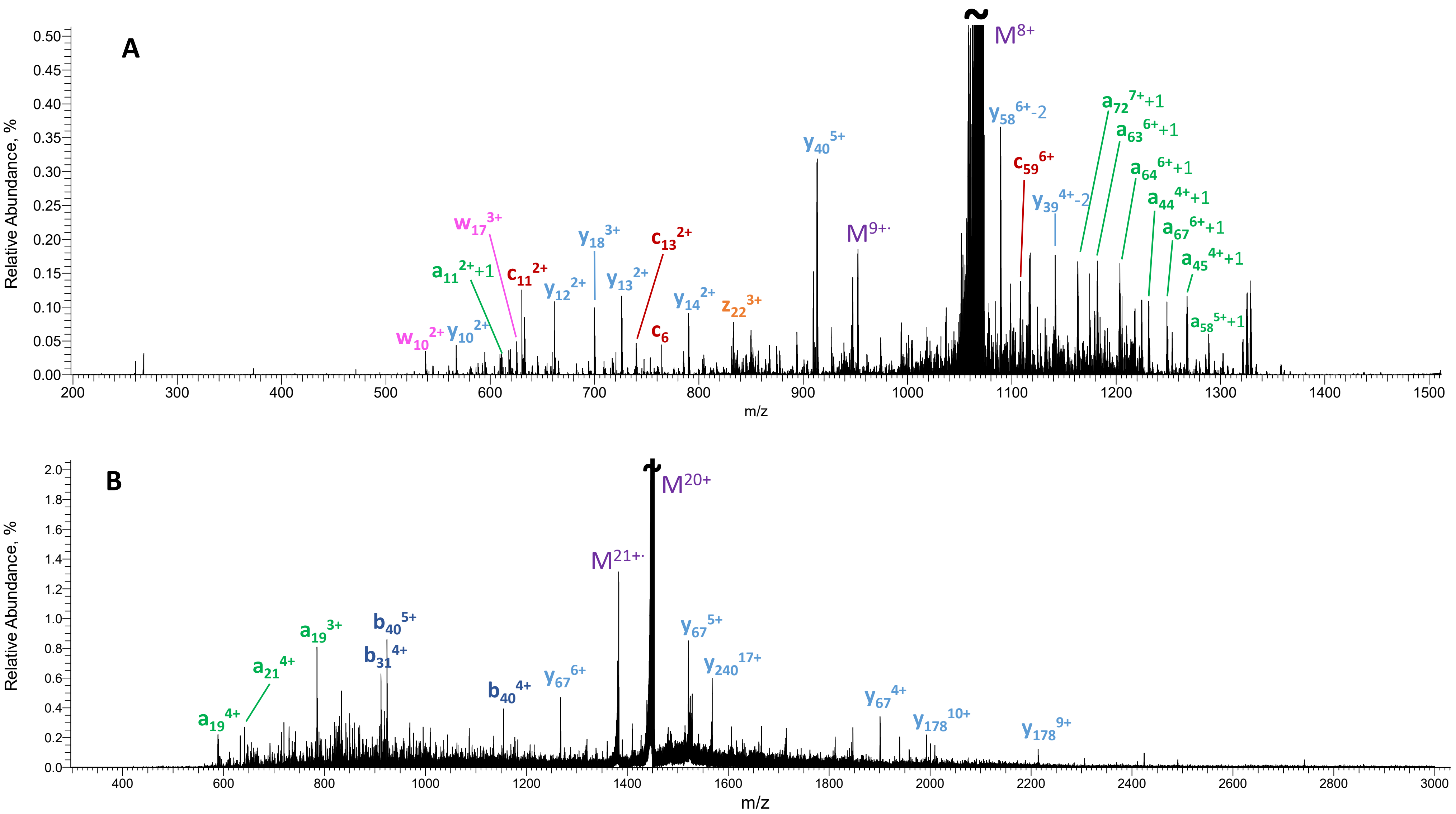

Figure S12. UVPD spectra of ubiquitin^{8+} (A) and [carbonic anhydrase]$^{20+}$ (B) acquired following three pulses of the 193 nm UV laser with the energy of 5 mJ per pulse. For clarity, only few selected fragments are annotated

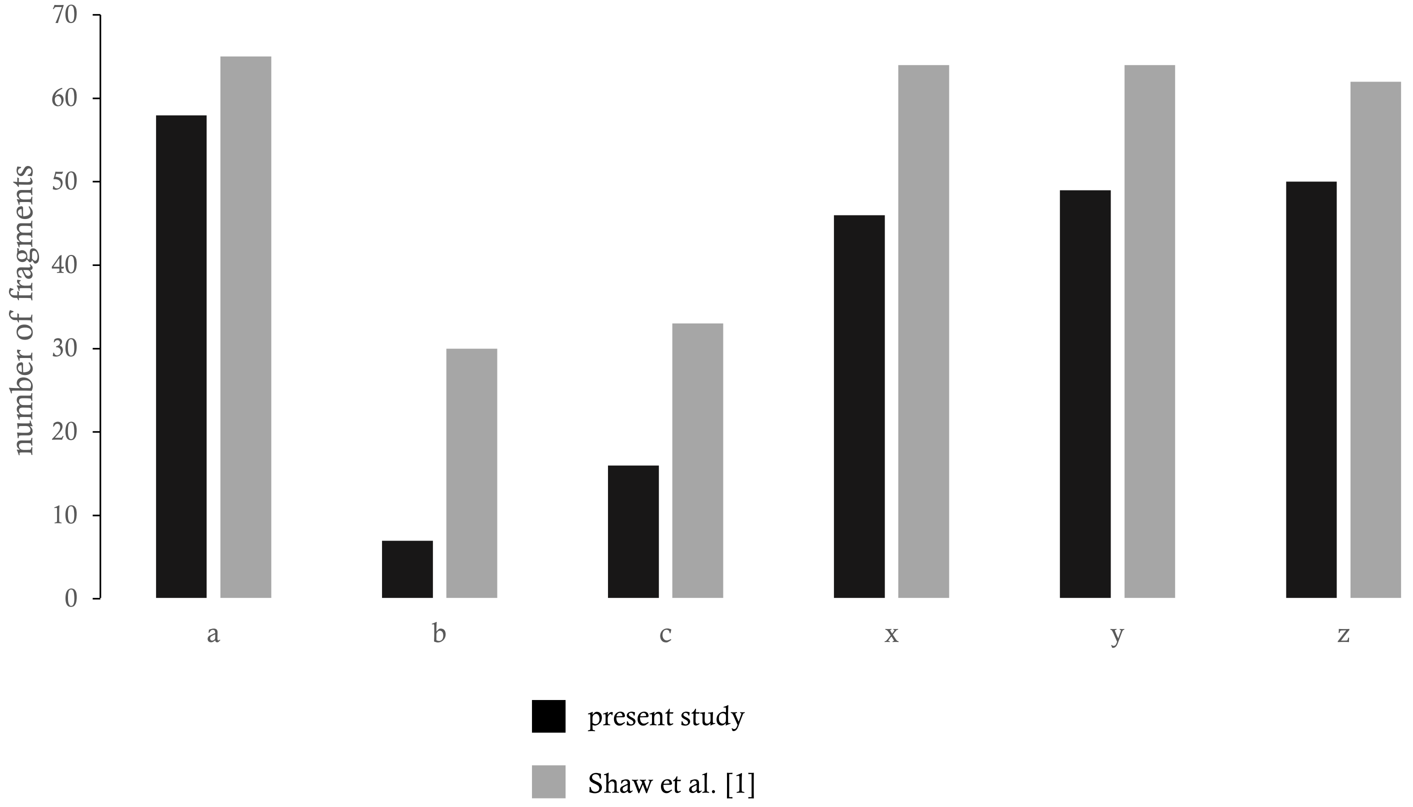

Figure S13. Number of fragments of different types identified in the UVPD experiments of ubiquitin^{8+} reported in this study (3 pulses, 5 mJ/pulse, black) and [Ref 1] (1 pulse, 1-4 mJ/pulse, grey)

[1] Shaw, J. B.; Li, W.; Holden, D. D.; Zhang, Y.; Griep-Raming, J.; Fellers, R. T.; Early, B. P.; Thomas, P. M.; Kelleher, N. L.; Brodbelt, J. S. Complete protein characterization using top-down mass spectrometry and ultraviolet photodissociation. *Journal of the American Chemical Society* **2013**, *135* (34), 12646-12651.

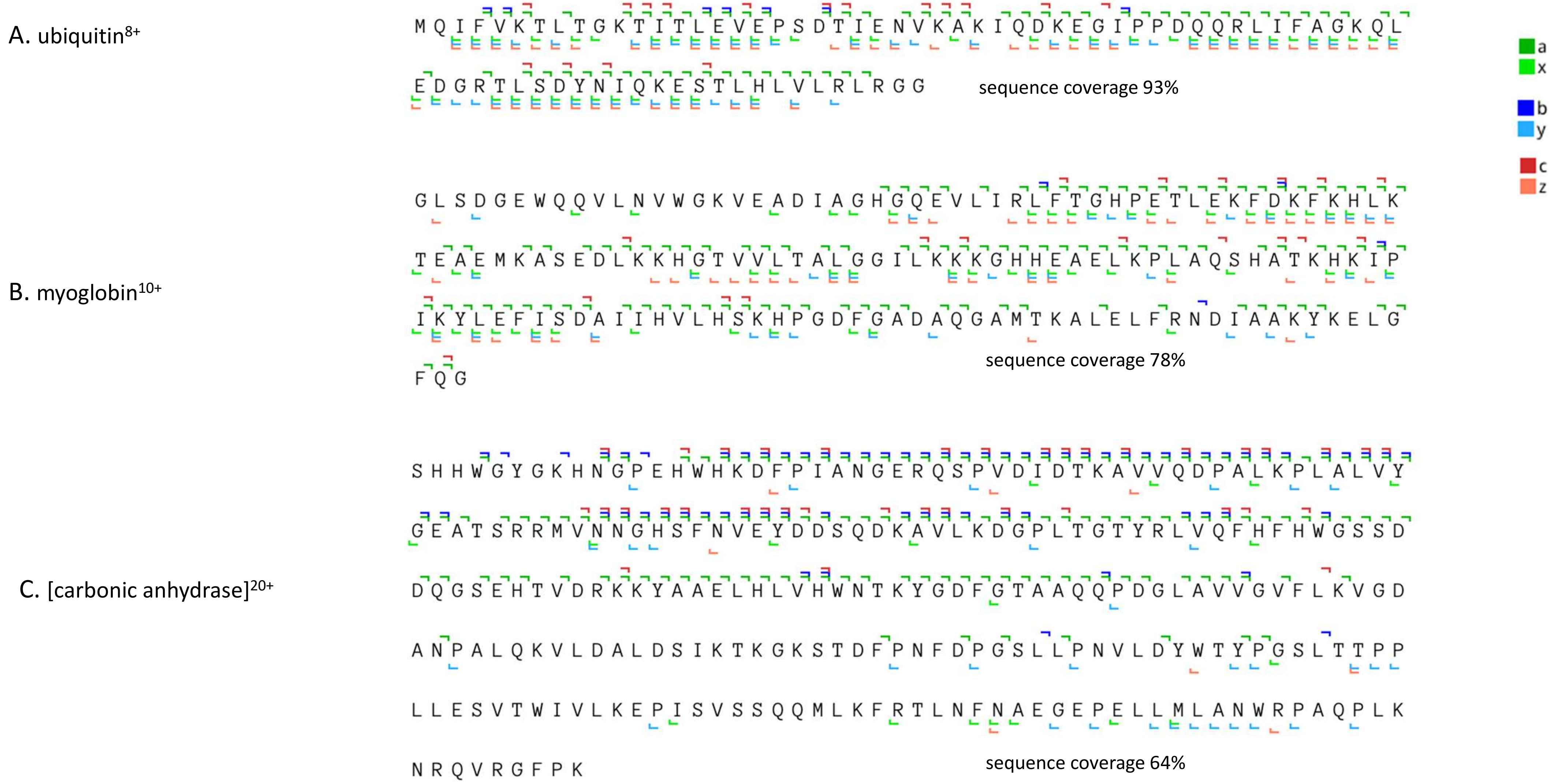

Figure S14. Fragment maps for UVPD experiments on ubiquitin^{8+} (A), myoglobin^{10+} (B), and [carbonic anhydrase]$^{20+}$ (C). The corresponding spectra were acquired following three pulses of the 193 nm UV laser with the energy of 5 mJ per pulse.

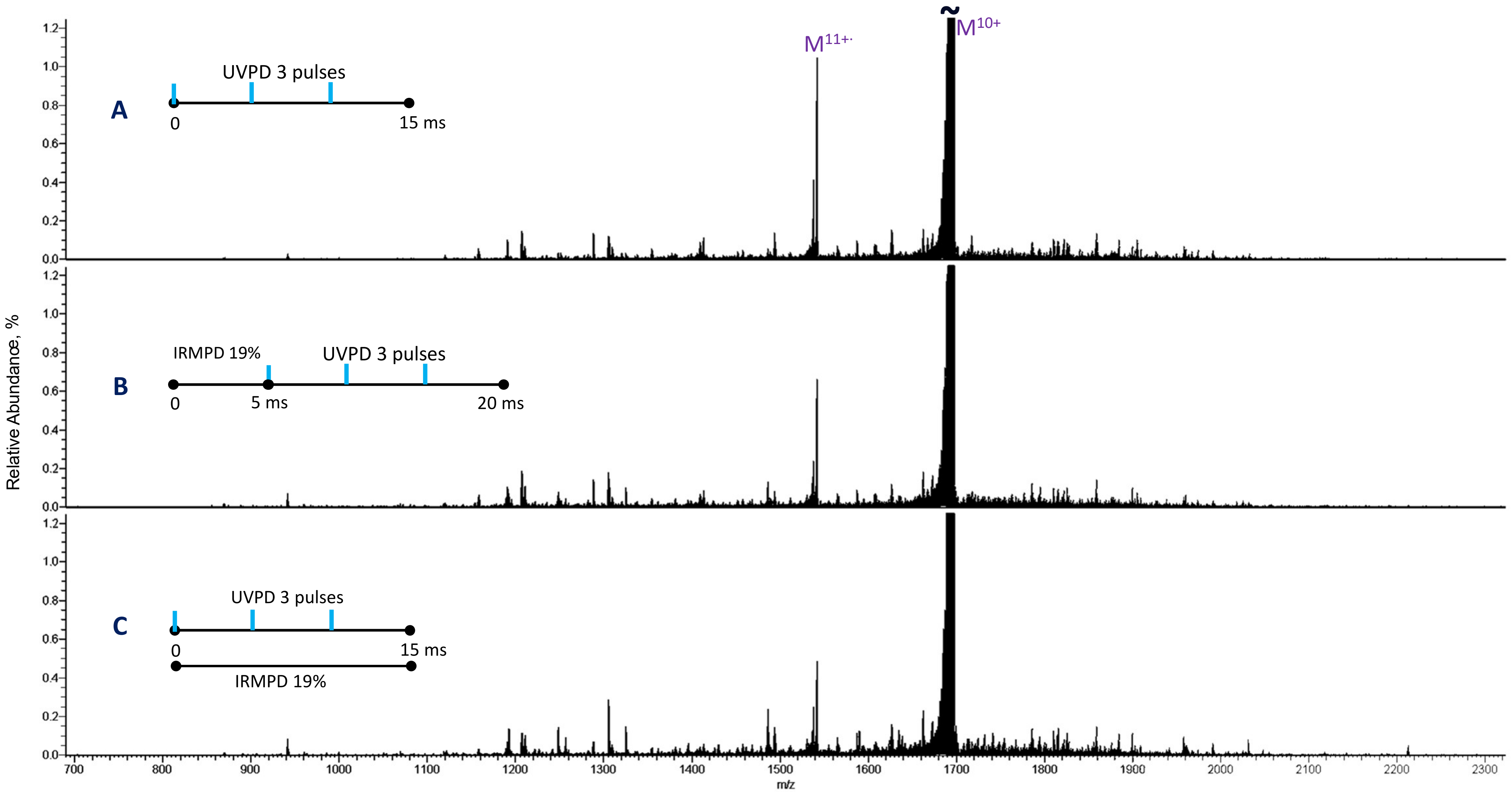

Figure S15. UVPD and IR-activated-UVPD spectra of myoglobin^{10+}. A) UVPD; B) IRMPD 5 ms (19 % laser duty cycle) followed by UVPD; C) continuous irradiation of precursor ions by IR (19 % laser duty cycle) for 15 ms during UVPD. In all experiments, three pulses of UV laser, 5 mJ/pulse were used for UVPD.

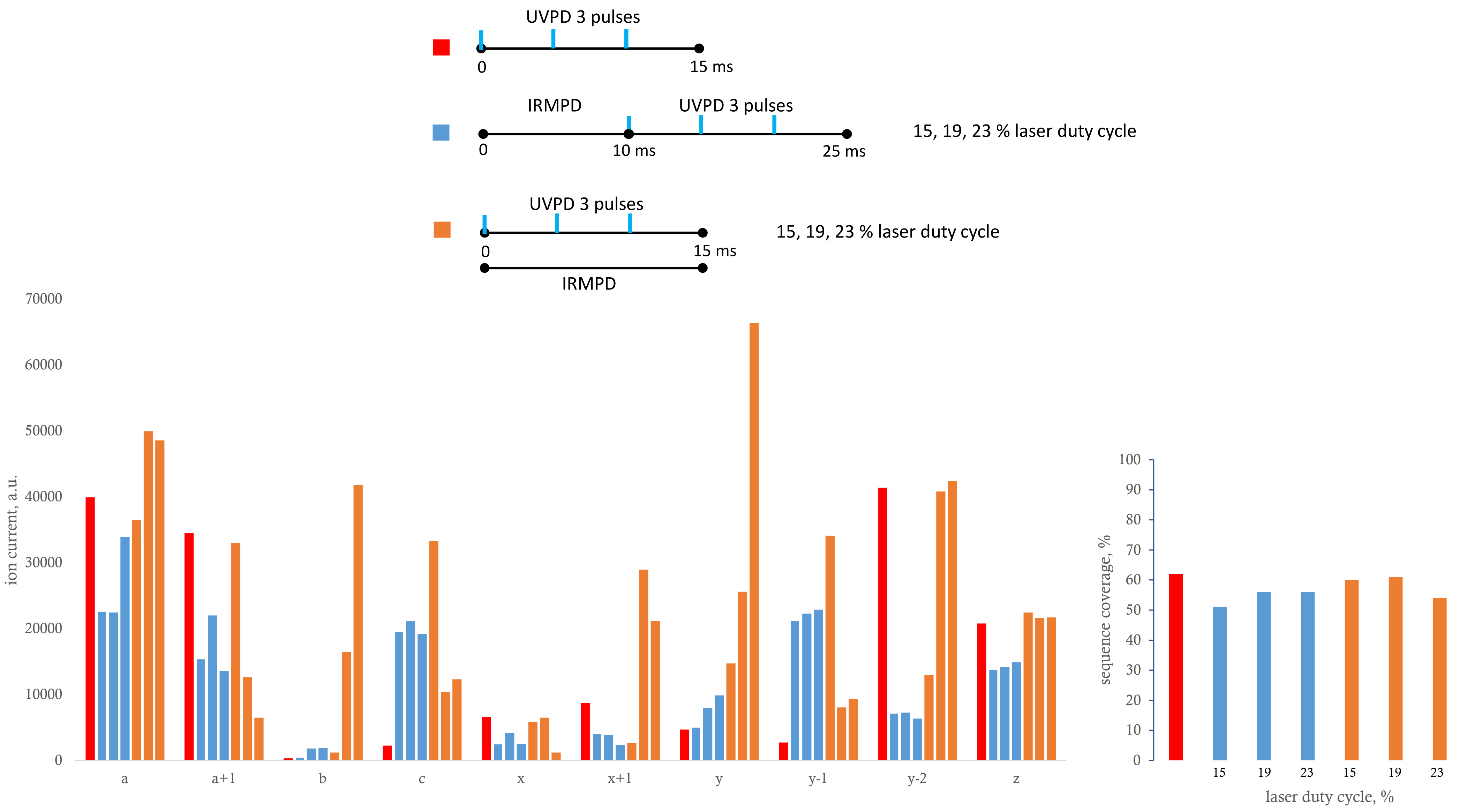

Figure S16. Ion currents of fragments of different types and sequence coverages identified in the IR-activated-UVPD experiments of myoglobin^{10+}.

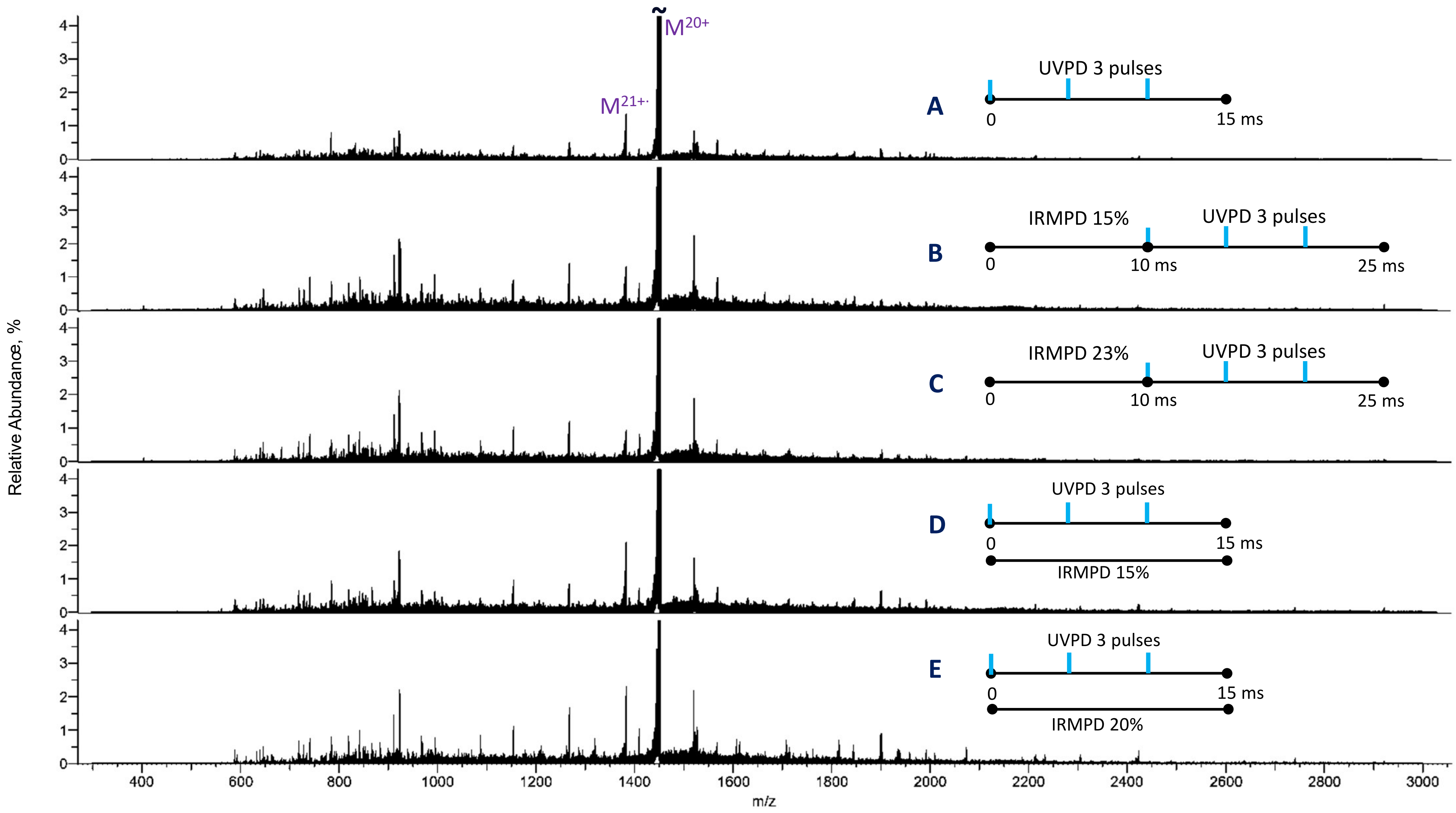

Figure S17. UVPD and IR-activated-UVPD spectra of [carbonic anhydrase]$^{20+}$. A) UVPD; B) IRMPD 10 ms (15 % laser duty cycle) followed by UVPD; C) IRMPD 10 ms (23 % laser duty cycle) followed by UVPD; D) continuous irradiation of precursor ions by IR (15 % laser duty cycle) for 15 ms during UVPD; E) continuous irradiation of precursor ions by IR (20 % laser duty cycle) for 15 ms during UVPD. In all experiments, three pulses of UV laser, 5 mJ/pulse were used for UVPD.

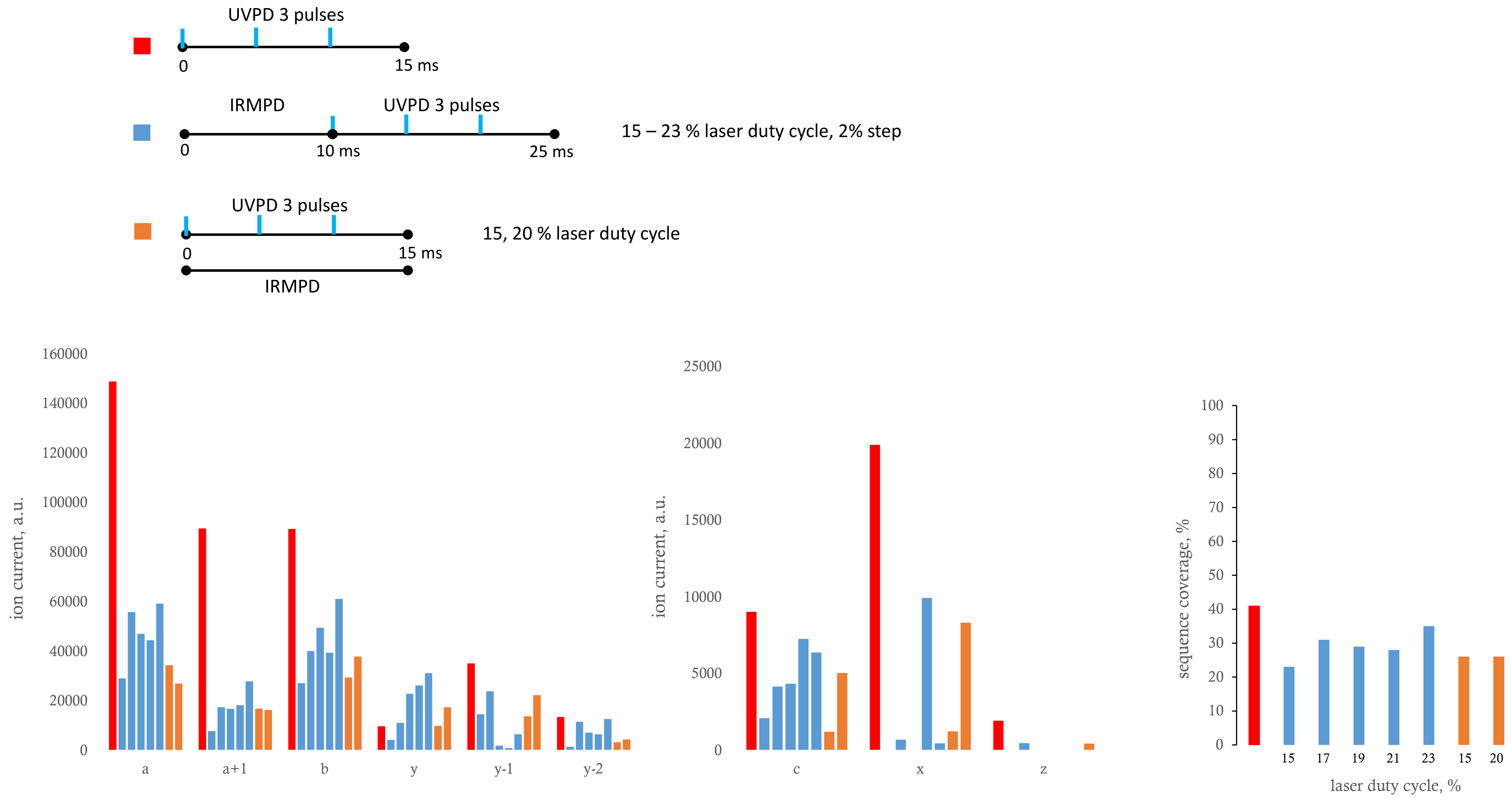

Figure S18. Ion currents of fragments of different types and sequence coverages identified in the IR-activated-UVPD experiments of [carbonic anhydrase]$^{20+}$.

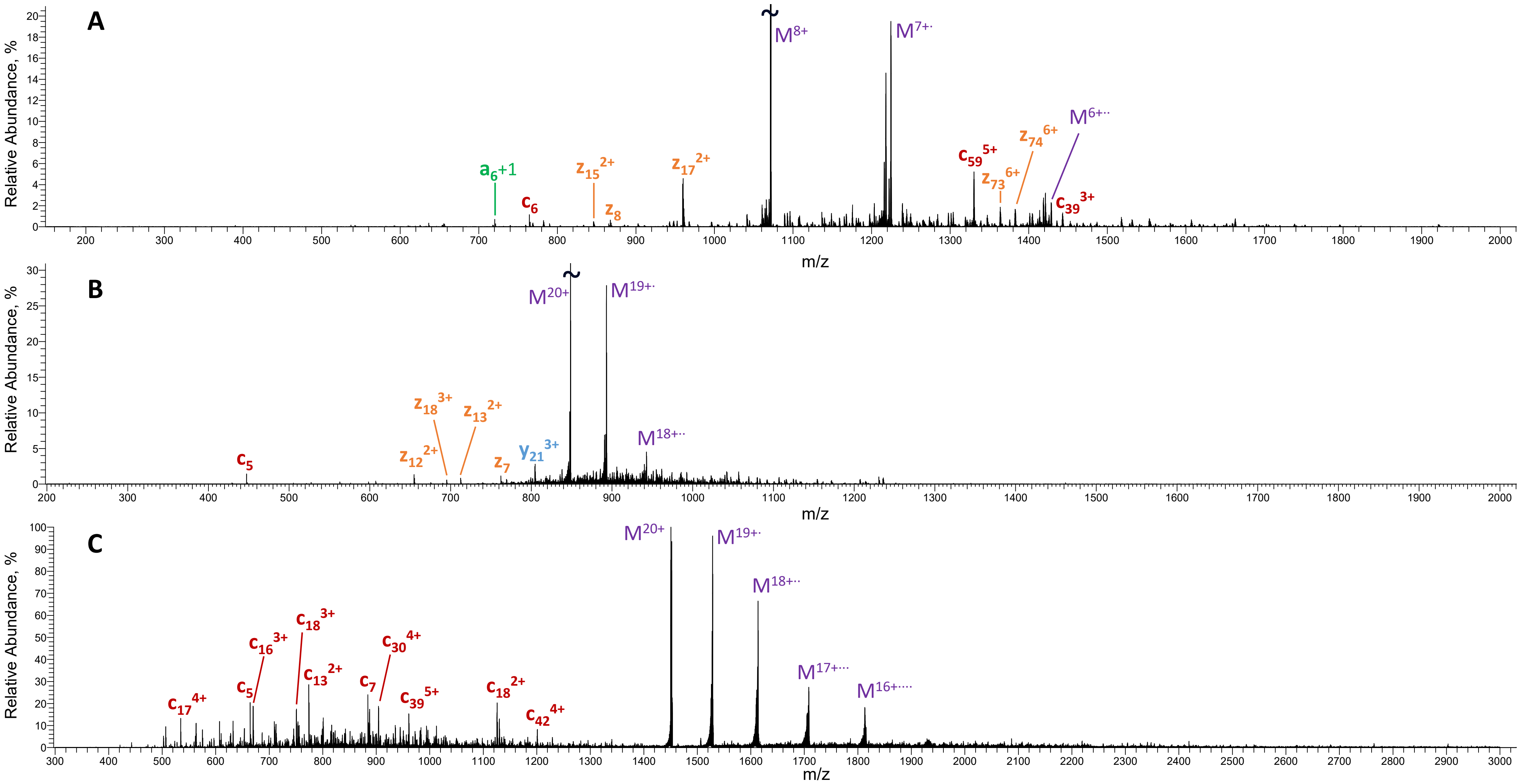

Figure S19. ECD spectra of ubiquitin^{8+} (A), myoglobin^{20+} (B), and [carbonic anhydrase]$^{20+}$ (C) acquired following 50 ms, 30 ms, and 20 ms of irradiation by 2 eV electrons, respectively. For clarity, only few selected fragments are annotated

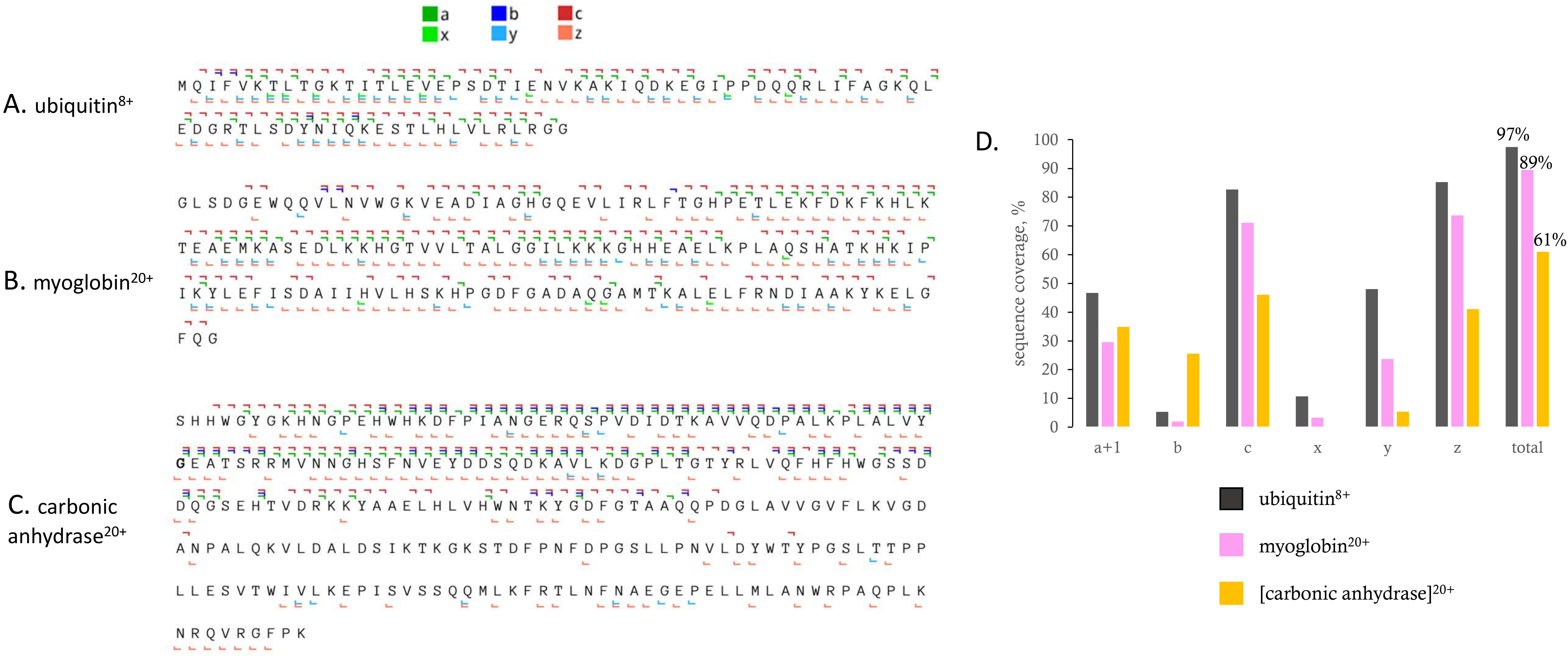

Figure S20. Fragment maps of ECD of A) ubiquitin^{8+} (50 ms irradiation), B) myoglobin^{20+} (30 ms irradiation), and C) [carbonic anhydrase]$^{20+}$ (20 ms irradiation). D) Sequence coverages by fragments of different types identified in the ECD experiments of ubiquitin^{8+} (50 ms irradiation), myoglobin^{20+} (30 ms irradiation), and [carbonic anhydrase]$^{20+}$ (20 ms irradiation). The energy of electrons in all experiments was 2 eV.

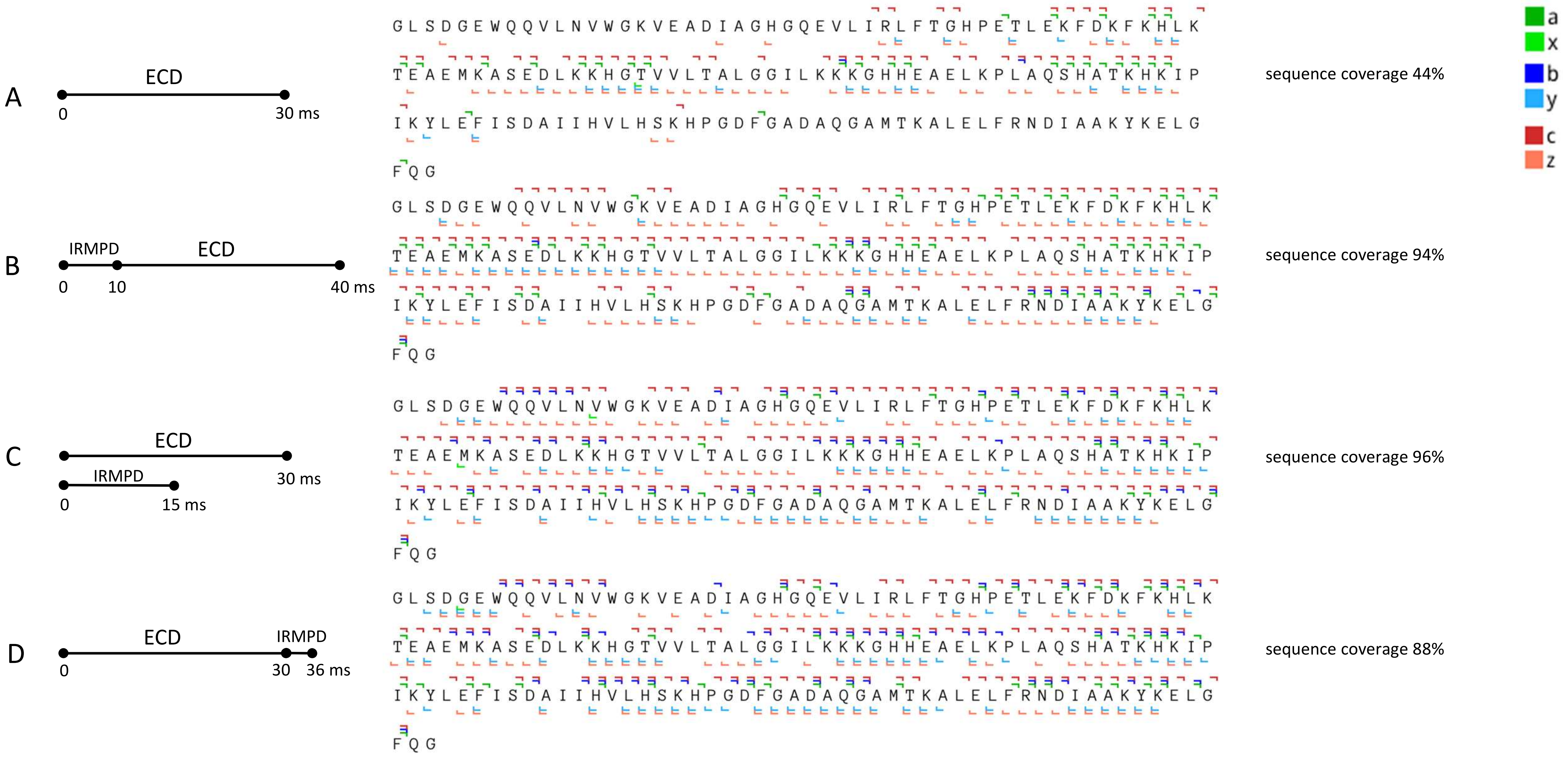

Figure S21. Fragment maps of ECD and IR-activated-ECD of myoglobin^{10+} as given in Figure 4. A) ECD 30 ms; B) IRMPD 10 ms (15 % laser duty cycle) followed by 30 ms ECD; C) IRMPD (14 % laser duty cycle) concurrently with ECD for 15 ms followed by 15 ms of ECD; D) ECD 30 ms followed by 6 ms IRMPD (19 % laser duty cycle). The energy of electrons in all experiments was 2 eV.

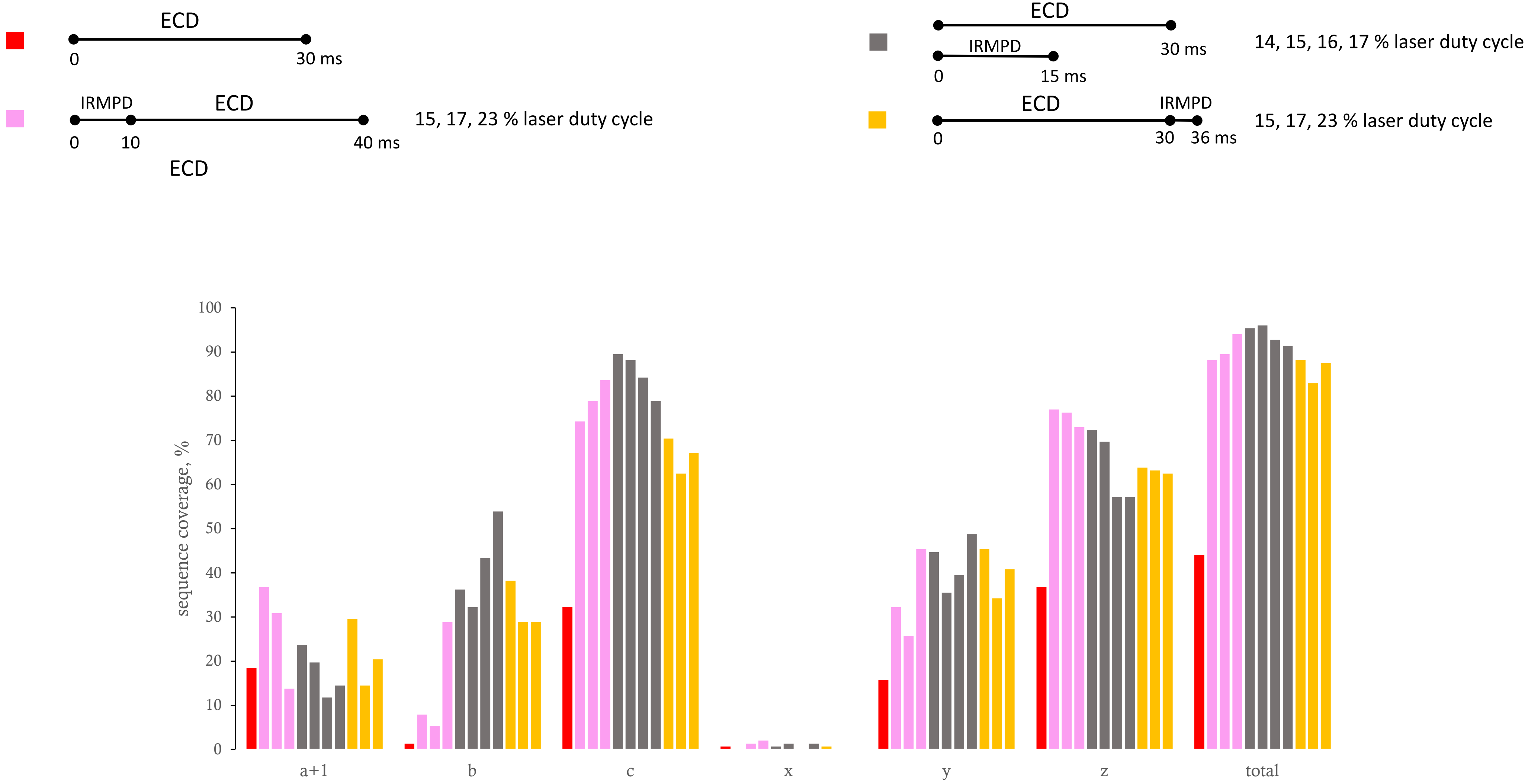

Figure S22. Sequence coverages by fragments of different types identified in the ECD and IR-activated-ECD experiments of myoglobin^{10+} as given in Figure 4.

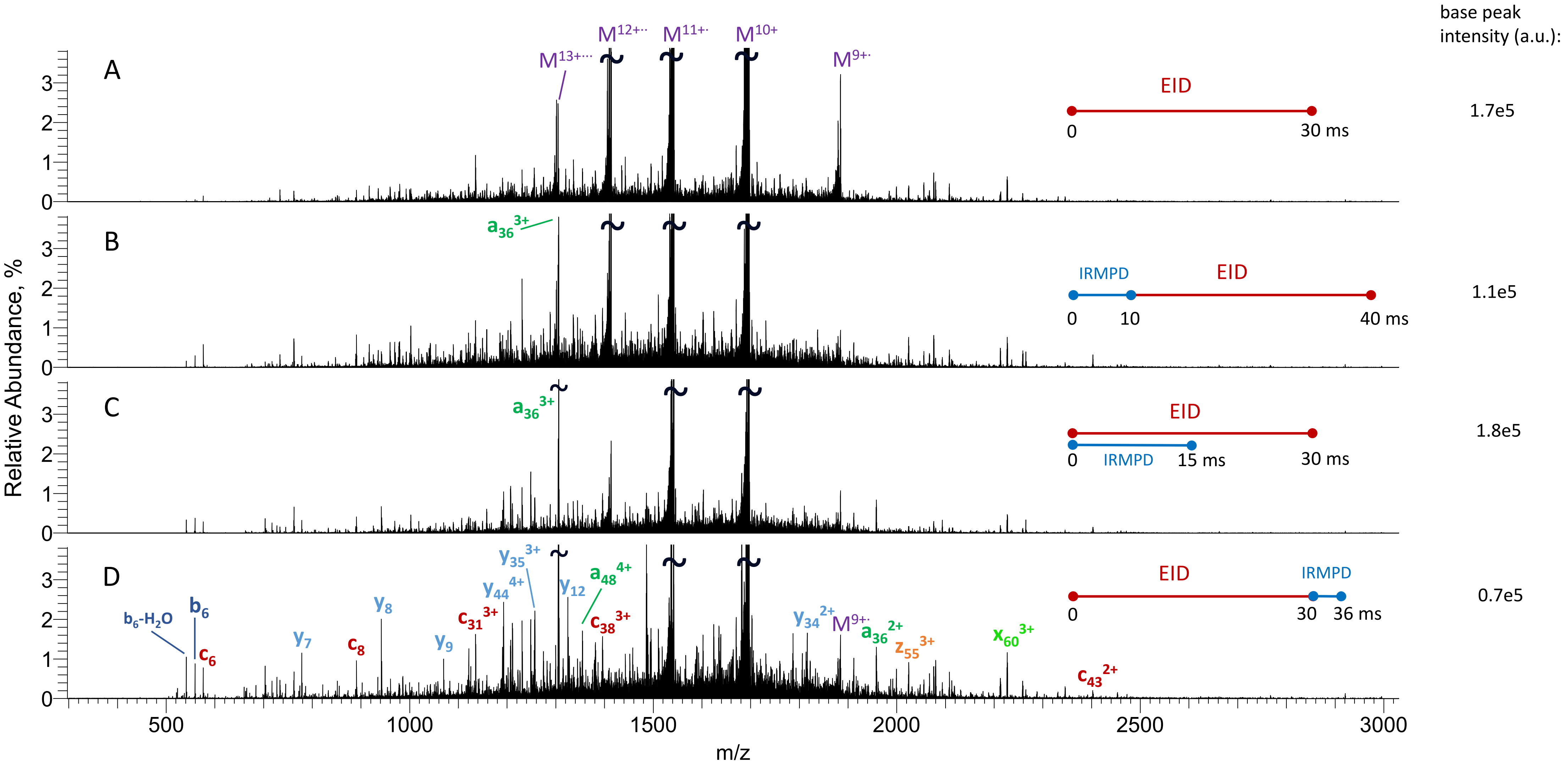

Figure S23. EID and IR-activated-EID spectra of myoglobin^{10+}. A) EID 30 ms; B) IRMPD 10 ms (15 % laser duty cycle) followed by 30 ms EID; C) simultaneous irradiation of precursor ions by IR light (14 % laser duty cycle) and electrons for 15 ms followed by 15 ms of EID; D) EID 30 ms followed by 6 ms IRMPD (19 % laser duty cycle). For clarity, only few selected fragments are annotated

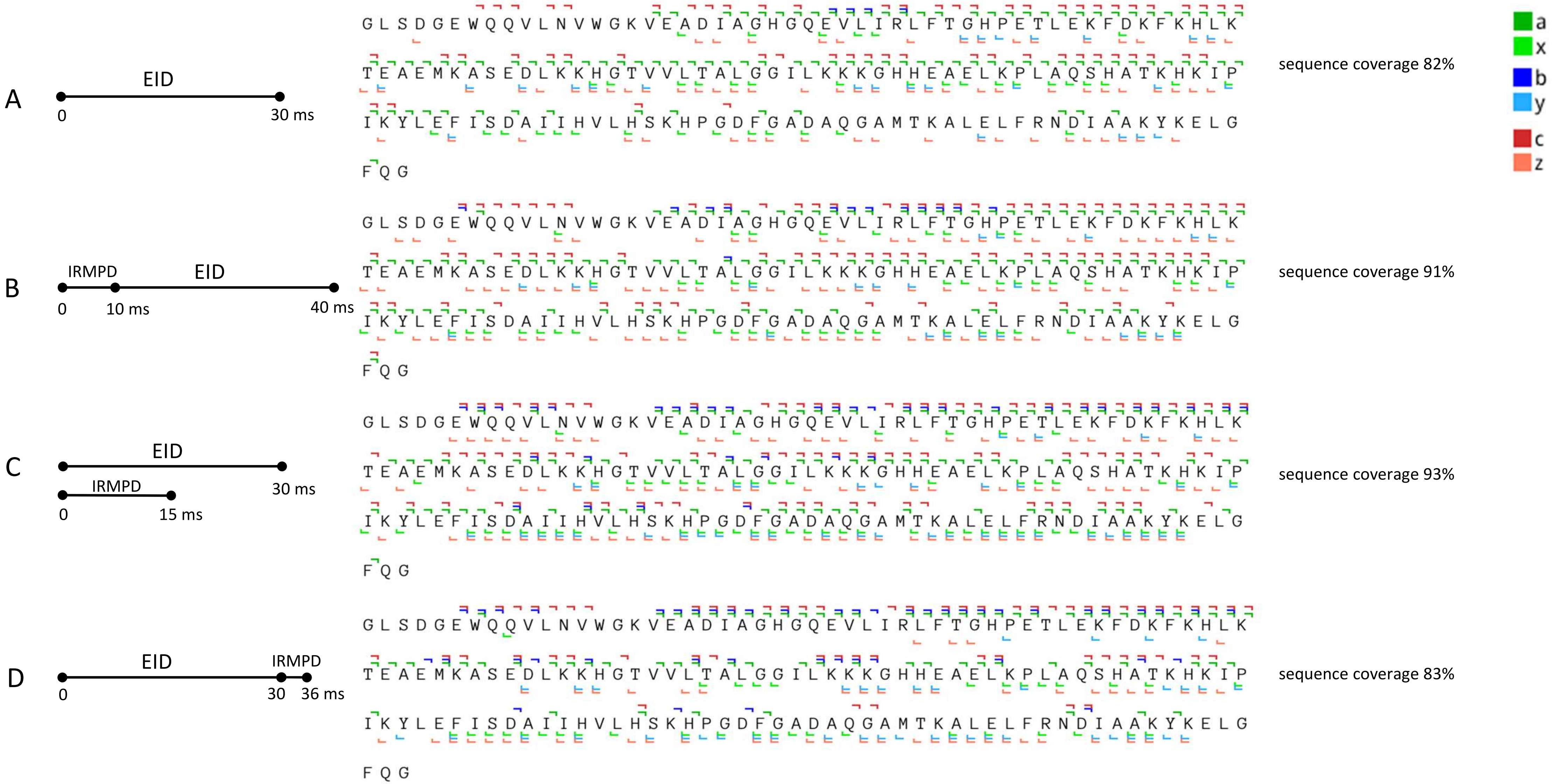

Figure S24. Fragment maps of EID and IR-activated-EID of myoglobin^{10+}. A) EID 30 ms; B) IRMPD 10 ms (15 % laser duty cycle) followed by 30 ms EID; C) simultaneous irradiation of precursor ions by IR light (14 % laser duty cycle) and electrons for 15 ms followed by 15 ms of EID; D) EID 30 ms followed by 6 ms IRMPD (19 % laser duty cycle). The energy of electrons in all experiments was 35 eV.

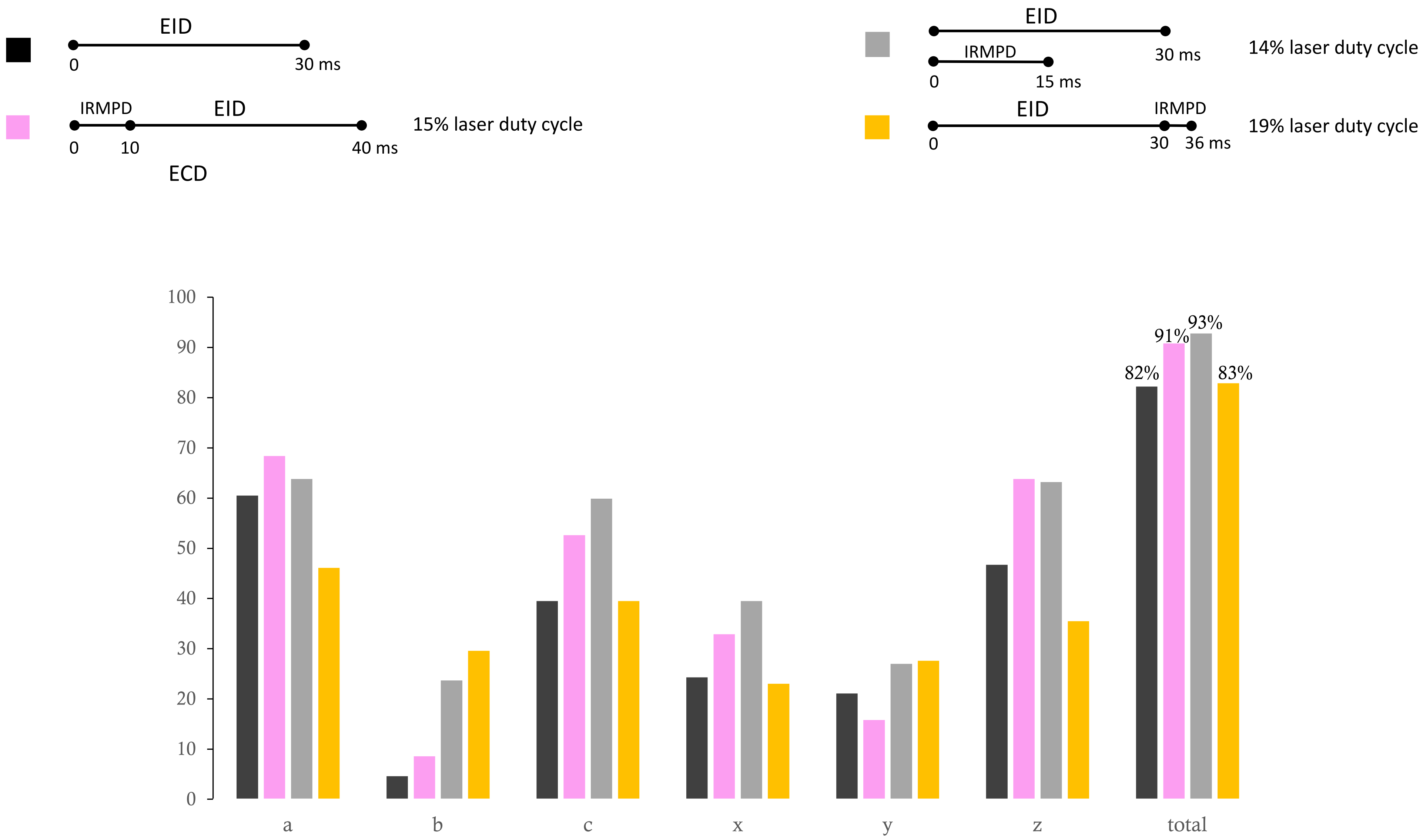

Figure S25. Sequence coverages by fragments of different types identified in the EID and IR-activated-EID experiments of myoglobin^{10+} as given in Figure S23.

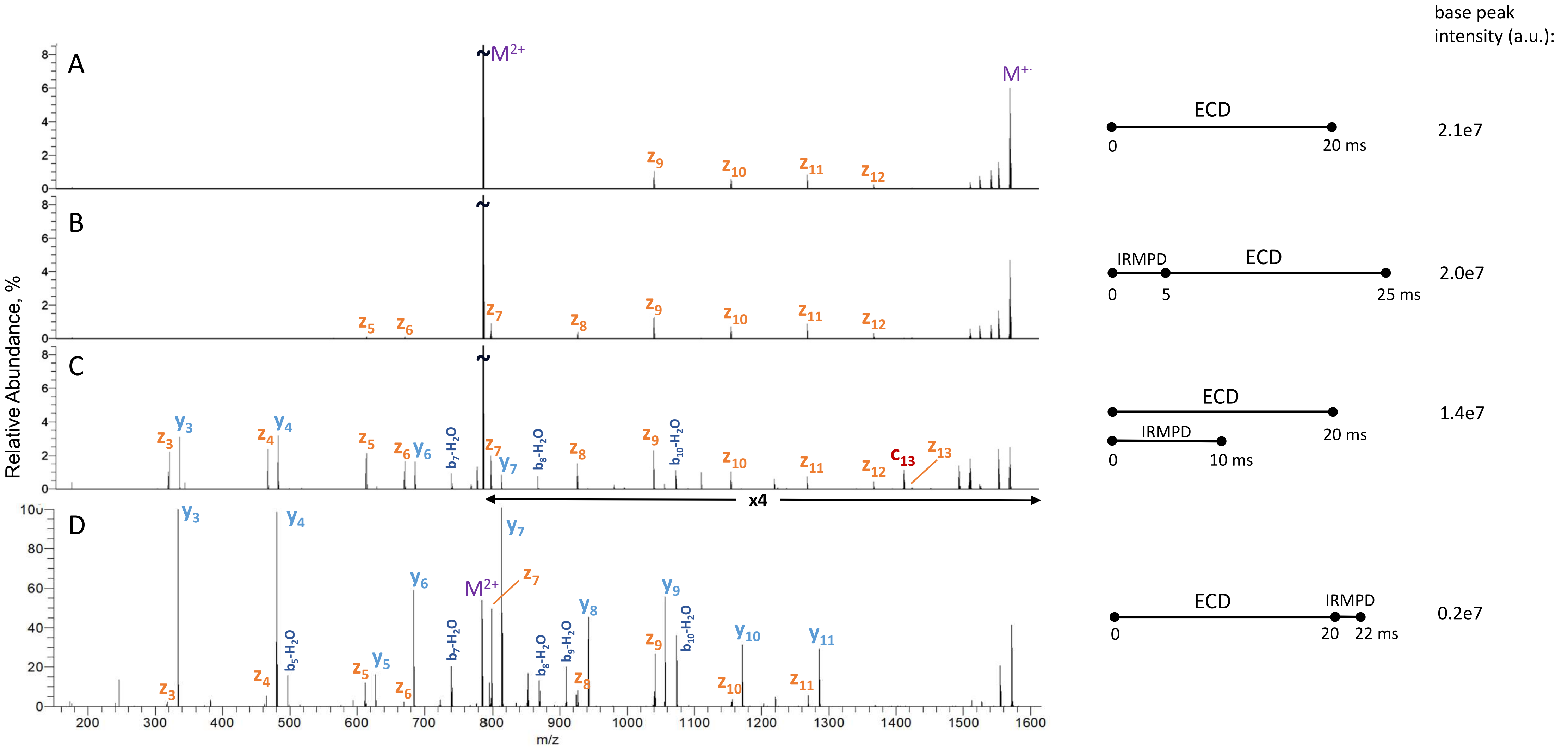

Figure S26. ECD and IR-activated-ECD spectra of [Glu-fibrinopeptide B]$^{2+}$. A) ECD 20 ms; B) IRMPD 5 ms (19 % laser duty cycle) followed by 20 ms ECD; C) simultaneous irradiation of precursor ions by IR (17 % laser duty cycle) and ECD for 10 ms followed by 10 ms of ECD; D) ECD 20 ms followed by 2 ms IRMPD (37 % laser duty cycle)

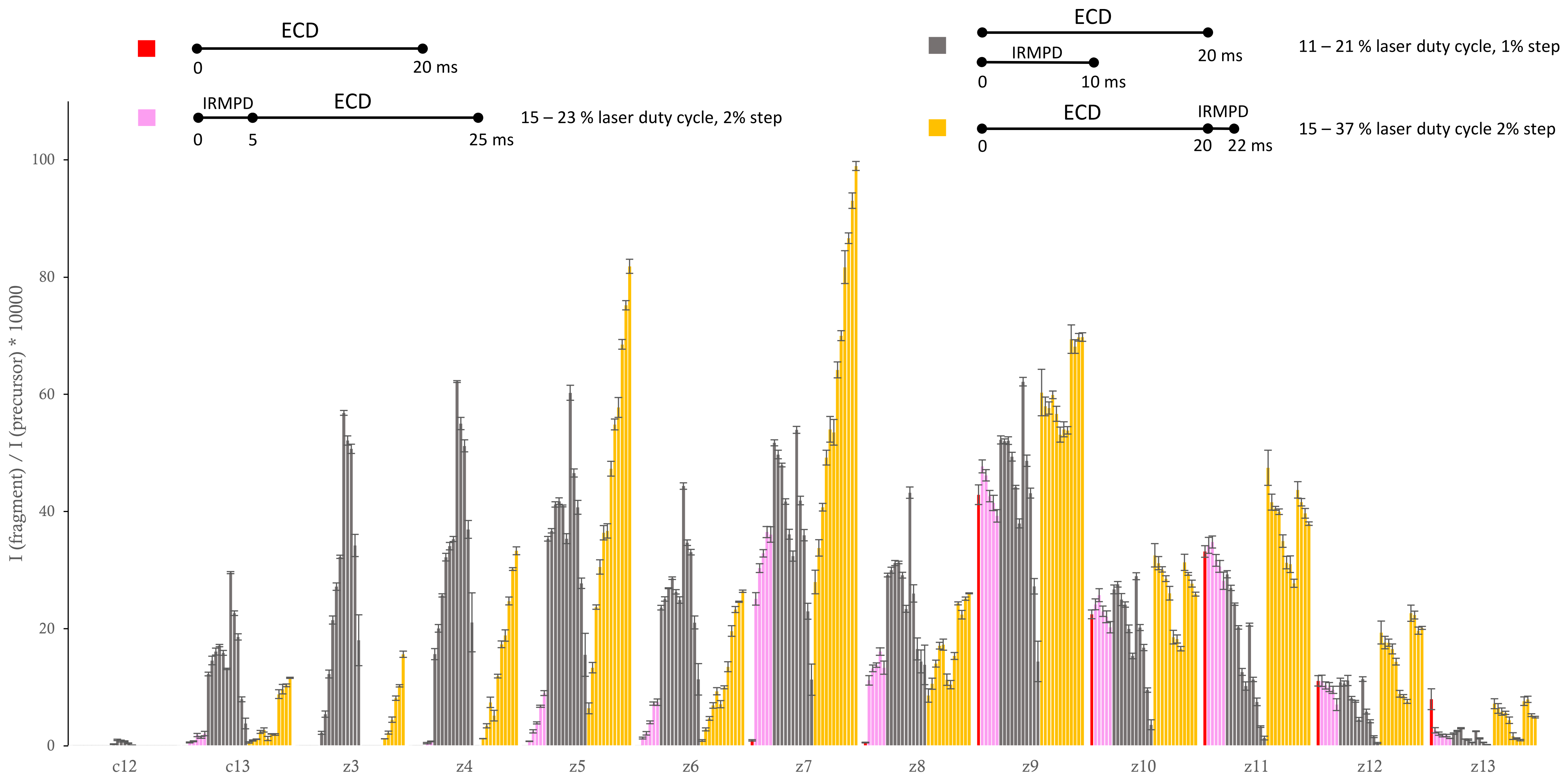

Figure S27. Distributions of normalised intensities of all *c* and *z* fragments identified in IR-activated-ECD of [Glu-fibrinopeptide B]$^{2+}$ for different power outputs of the IR laser. The experiment was run in triplicate. Each data bar represents an average of a triplicate with standard deviations given as error bars. The intensities of fragments in each acquisition were normalised to the intensity of the isolated precursor in MS1.

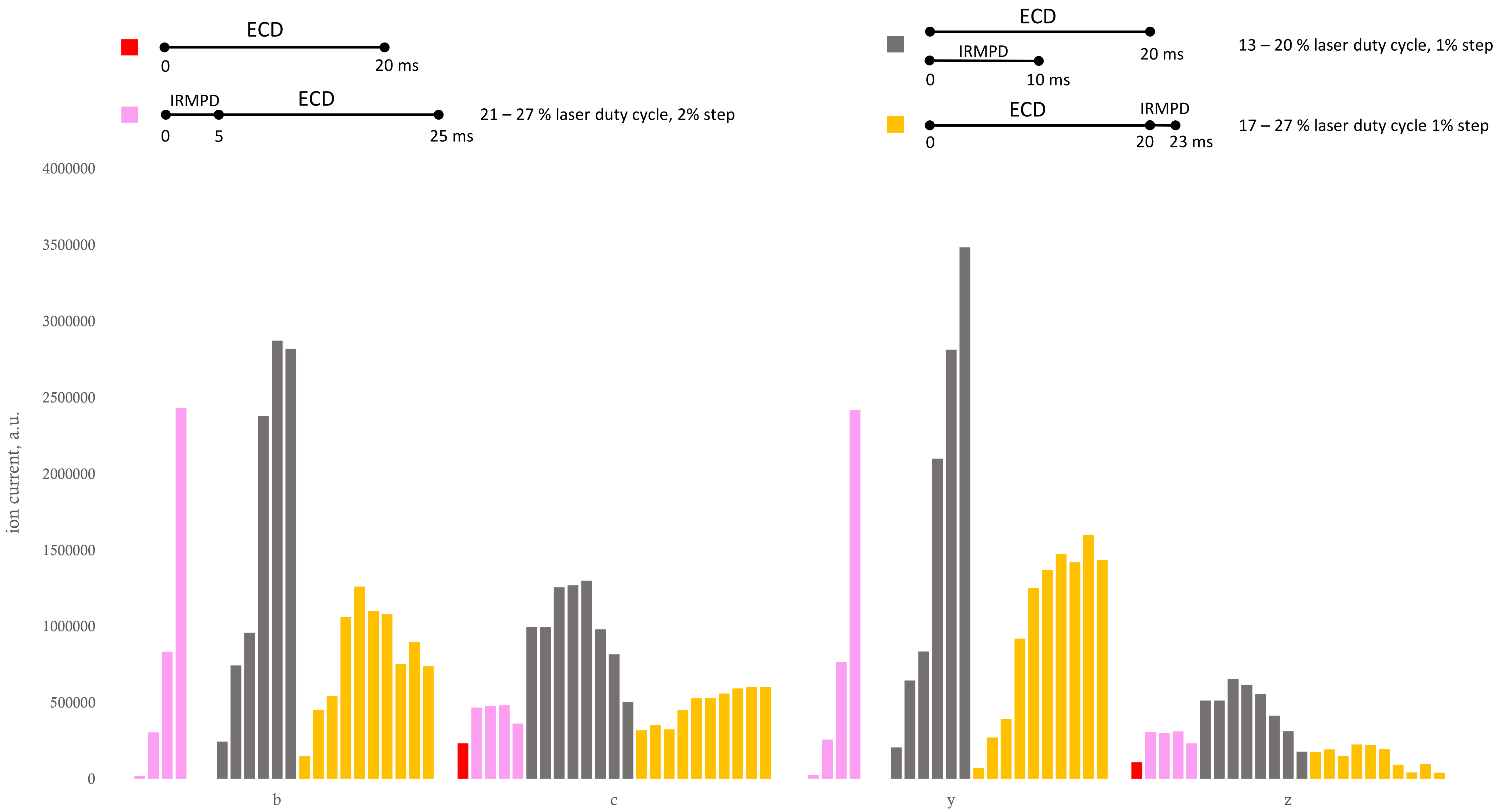

Figure S28. Summed ion currents of *a*,*b*,*c*,*y*,*z* fragments identified in IR-activated-ECD of triply charged chain B of insulin for different power outputs of the IR laser.

Table S1. Details about analytes used in the experiments

analyte	sequence	modification	concentration/solvent	monoisotopic mass
bradykinin	RPPGFSPFR	-	1 µM in<br>50:25:24:1 v/v ACN:MeOH:H_2O:acetic acid	1059.56
Glu-fibrinopeptide B	EGVNDNEEGFFSAR	-	0.1 µM in<br>49.5:49.5:1 ACN:water:formic acid	1569.67
insulin chain B	FVNQHLCGSHLVEALYLVCGERGFFYTPKA	Trioxidation of Cys residues	1 µM in<br>49.5:49.5:1 ACN:water:formic acid	3493.64
ubiquitin	MQIFVKTLTGKTITLEVEPSDTIENVKAKIQD<br>KEGIPPDQQRLIFAGKQLEDGRTLSDYNIQKE<br>STLHLVLRLRGG	-	0.5 µM in<br>49.5:49.5:1 ACN:water:acetic acid	8559.62
myoglobin	GLSDGEWQQVLNVWGKVEADIAGHGQEVLI<br>RLFTGHPETLEKFDKFKHLKTEAEMKASEDL<br>KKHGTVVLTALGGILKKKGHHEAELKPLAQS<br>HATKHKIPIKYLEFISDAIIHVLHSKHPGDFGA<br>DAQGAMTKALELFRNDIAAKYKELGFQG	-	0.1 µM in<br>49.5:49.5:1 ACN:water:formic acid	16983.98
carbonic anhydrase	SHHWGYGKHNGPEHWHKDFPIANGERQSPV<br>DIDTKAVVQDPALKPLALVYGEATSRRMVNN<br>GHSFNVEYDDSQDKAVLKDGPLTGTYRLVQF<br>HFHWGSSDDQGSEHTVDRKKYAAELHLVHW<br>NTKYGDFGTAAQQPDGLAVVGVFLKVGDANP<br>ALQKVLDALDSIKTKGKSTDFPNFDPGSLLPN<br>VLDYWTYPGSLTTPPLLESVTWIVLKEPISVS<br>SQQMLKFRTLNFNAEGEPELLMLANWRPAQ<br>PLKNRQVRGFPK	N-term acetylation	10 µM in<br>49.5:49.5:1 ACN:water:formic acid	29006.68

Table S2. Types of fragments searched in the spectra using the in-house software and MS-TAFI

**fragmentation technique**	**types of fragments searched (in-house software)**	**types of fragments searched (MS-TAFI)**
IRMPD	b, y	b, y (CID option)
UVPD<br>IR-activated-UVPD	a, a+1, b, b+1, b+2, c, x, x+1, y, y-1, y-2, z	a, a+1, b, c, x, x+1, y, y-1, y-2, z (UVPD option)
ECD	a, a+1, c, c+1, c-1, y, z, z+1, z-1	a, a+1, b, c, x, x+1, y, y-1, y-2, z (UVPD option)
IR-activated-ECD	a, a+1, b, c, c+1, c-1, y, z, z+1, z-1	a, a+1, b, c, x, x+1, y, y-1, y-2, z (UVPD option)
EID<br>IR-activated-EID	a, a+1, b, c, c-1, c+1, x, x+1, y, z, z-1, z+1	a, a+1, b, c, x, x+1, y, y-1, y-2, z (UVPD option)
